# The forgotten exotic tapeworms: a review of uncommon zoonotic Cyclophyllidea

**DOI:** 10.1017/S003118202000013X

**Published:** 2020-04

**Authors:** Sarah G. H. Sapp, Richard S. Bradbury

**Affiliations:** 1 Parasitic Diseases Branch, Division of Parasitic Diseases and Malaria, Centers for Disease Control and Prevention, 1600 Clifton Rd, Atlanta, Georgia, USA; 2 School of Health and Life Sciences, Federation University Australia, 100 Clyde Rd, Berwick, Victoria, AUS 3806, Australia

**Keywords:** *Bertiella*, Cestodes, Cyclophyllidea, *Dipylidium*, *Inermicapsifer*, *Mesocestoides*, *Raillietina*, Zoonoses

## Abstract

As training in helminthology has declined in the medical microbiology curriculum, many rare species of zoonotic cestodes have fallen into obscurity. Even among specialist practitioners, knowledge of human intestinal cestode infections is often limited to three genera, *Taenia*, *Hymenolepis* and *Dibothriocephalus*. However, five genera of uncommonly encountered zoonotic Cyclophyllidea (*Bertiella*, *Dipylidium*, *Raillietina*, *Inermicapsifer* and *Mesocestoides*) may also cause patent intestinal infections in humans worldwide. Due to the limited availability of summarized and taxonomically accurate data, such cases may present a diagnostic dilemma to clinicians and laboratories alike. In this review, historical literature on these cestodes is synthesized and knowledge gaps are highlighted. Clinically relevant taxonomy, nomenclature, life cycles, morphology of human-infecting species are discussed and clarified, along with the clinical presentation, diagnostic features and molecular advances, where available. Due to the limited awareness of these agents and identifying features, it is difficult to assess the true incidence of these ‘forgotten’ cestodiases as clinical misidentifications are likely to occur. Also, the taxonomic status of many of the human-infecting species of these tapeworms is unclear, hampering accurate species identification. Further studies combining molecular data and morphological observations are necessary to resolve these long-standing taxonomic issues and to elucidate other unknown aspects of transmission and ecology.

## Introduction

The order Cyclophyllidea includes the ‘classic’ tapeworms and represents the largest cestode order, with over 3000 named species (Mariaux *et al*., 2017). Perhaps the most familiar members are the intestinal tapeworms commonly infecting humans; *Taenia solium*, *T. saginata*, *T. asiatica* and *Hymenolepis nana.* Agents of human cestode infections other than these are seldom discussed, if even covered at all within medical educational curricula and textbooks. However, several other cestode genera exist that can colonize the intestinal tract human hosts and produce patent infections – but these also suffer from a great sparsity of clinical and diagnostic information, research and modern interest. In many clinical settings, even generic identification of tapeworm infections is either never performed or automatically ascribed to well-known agents (e.g. *Taenia* spp.) without a detailed examination of parasite proglottid, scolex or egg morphology. This further obscures the true diversity and occurrence of these zoonotic cestodiases.

**Fig. 1.**
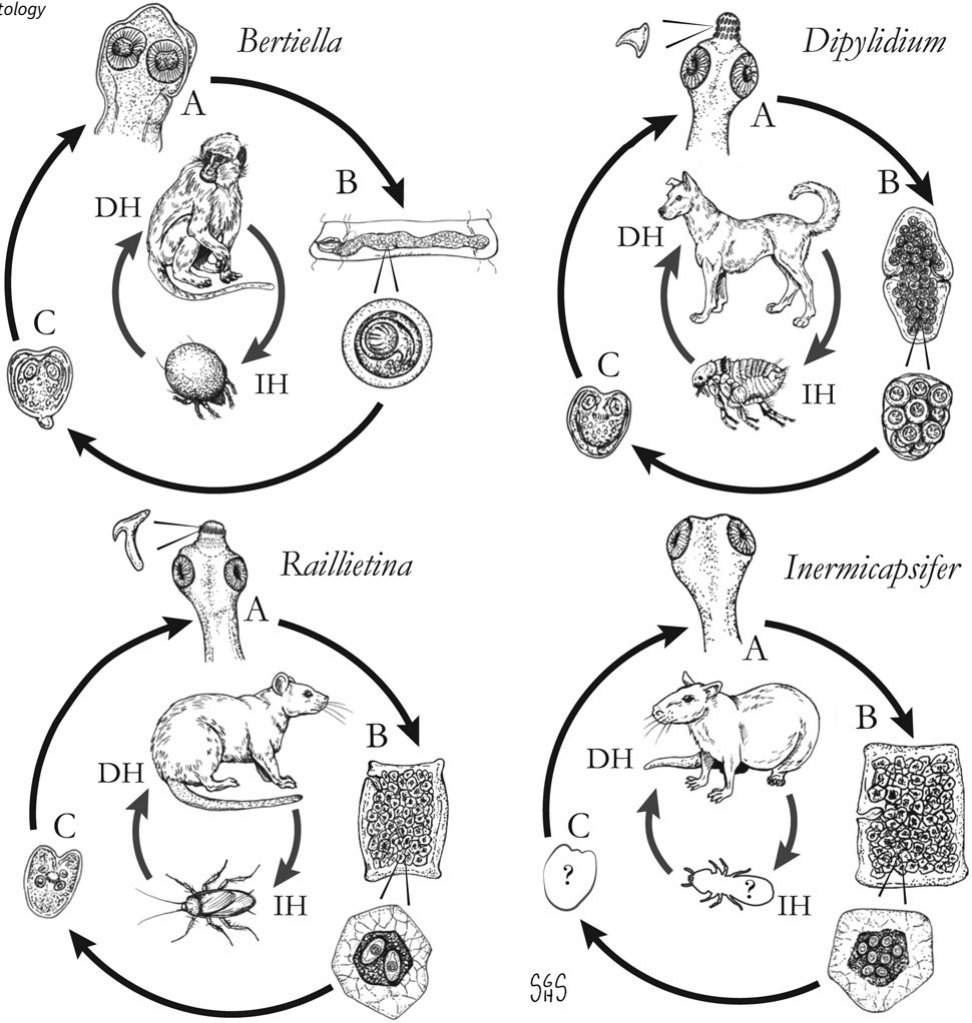
Generalized life cycles for *Bertiella*, *Dipylidium*, *Raillietina* and *Inermicapsifer* spp. Cestode stages shown on the outside: (A) scolex of an adult; (B) gravid proglottids with egg(s); (C) cysticercoid; representative definitive hosts (DH) and intermediate hosts (IH) on the inside (Drawings by SGH Sapp).

The major zoonotic, non-taeniid and non-hymenolepid cyclophyllidean genera that cause intestinal infections are *Bertiella*, *Dipylidium*, *Raillietina*, *Inermicapsifer* and *Mesocestoides* (Table 1) (Belding, 1965; Beaver *et al*., 1984). All of these genera are normally associated with a variety of mammalian definitive hosts – primates for *Bertiella*, carnivores for *Dipylidium* and *Mesocestoides*, and rodents for *Raillietina* and *Inermicapsifer* (Chandler and Pradatsundarasar, 1957; Goldsmid, 1964; James, 1968; Beaver *et al*., 1984). They undergo indirect life cycles typical of most cestodes, though several aspects of transmission or descriptions of life stages are unknown or poorly understood (Fig. 1). Assessing the true incidence of these infections in humans is difficult due to the relative scarcity of reports and potential misidentifications. Singular reports of other ‘unusual’ cyclophyllidean genera in human also exist [e.g. *Drepanidotaenia* (Belding, 1965), *Mathevotaenia* (Lamom and Greer, 1986), *Moniezia* (El-Shazly *et al*., 2004)]. However, their validity is not easy to evaluate, so this review will focus on the five genera. For each genus, life history, clinically-relevant taxonomy, morphological features and diagnosis, features of known human cases and treatment will be discussed.

**Table 1.** Overview of characteristics of ‘unusual’ zoonotic agents within order Cyclophyllidea

	*Bertiella*	*Dipylidium*	*Raillietina*	*Inermicapsifer*	*Mesocestoides*
Taxonomic authority	Stiles and Hassall (1902)	Leuckart 1863	Fuhrmann 1920	Janicki 1910	Valliant 1863
Family (Subfamily)	Anoplocephalidae (Anoplocephalinae)	Dipylidiidae	Davaineidae (Davaineinae)	Anoplocephalidae^a^ (Inermicapsiferinae)	Mesocestoididae (Mesocestoidinae)
Reported zoonotic species	*B. mucronata* *B. studeri* *B. satyri* ?	*D. caninum*	*R. celebensis* *R. demerariensis* *R. siriraji* *R. madagascarensis*^b^	*I. madagascariensis (* *=* *cubensis*) ( = *arvicanthidis)*	*M. lineatus* *M. variabilis*
Usual definitive host(s)	Primates: Old World (*B. studeri*) and New World (*B. mucronata*)	Canids and felids; most common in domestic species	Rodents (known zoonotic species)^c^, rarely primates	Rodents: Natal multimammate rat (*Mastomys natalensis*), Gambian pouched rat (*Cricetomys gambianus*); Rock hyrax (*Procavia capensis*)	Terrestrial carnivores; e.g. canids, felids, procyonids, mustelids, some marsupials
Usual intermediate host(s)	Oribatid mites	Fleas (*Ctenocephalides* spp.), dog louse (*Trichodectes canis*)	Insects; ants, cockroaches, beetles, etc.	Unknown	First intermediate host unknown^a^; Second intermediate hosts are numerous mammals, birds, reptiles, amphibians
Geographic range	*B. mucronata:* South America; *B. studeri*: South East Asia, South Asia, Middle-East, Sub-Saharan Africa, Mauritius, St. Kitts.	Cosmopolitan	Likely cosmopolitan; human infections in Southeast Asia, Australia, Hawaii, South America	Sub-Saharan Africa and West Indies	Cosmopolitan in animal hosts; most human cases from East Asia, USA
Scolex	Four large cup shaped suckers; rostellum and hooks absent -*B. satyri* has a knob-like projection on the apex of its scolex.	Four round to oval suckers; Rostellum armed with multiple transverse rows of rose thorn-shaped hooks.	Four round suckers with small spines; rostellum armed with alternating small/large hammer-shaped hooks; rostellar sac lined with small accessory spines.	Four round, aspinous suckers; rostellum and hooks absent.	Four large, round suckers; rostellum and hooks absent.
Proglottids^d^ (mm)	6–15 × 1–3 mm; much broader than tall; lateral genital pore.	7 × 3 mm; Highly motile and variable in length, elongate, rounded, ‘cucumber seed’ shape; two opposing genital pores situated on the median lateral margins.	3 × 2 mm; Barrel-shaped, containing numerous egg capsules (100–400), which can obscure genital organs; genital pore on anterior portion of lateral margin.	3 × 2.5 mm; Numerous egg capsules; genital pore on median lateral margin.	2.25 × 1.3 mm; Barrel-shaped, with parauterine organ; genital pore opens centrally on ventral surface.
Eggs	35–62 *μ*m; Spherical, with a filamentous vitelline membrane; well-developed pyriform apparatus surrounding oncospheres.	40–50 *μ*m; *Hymenolepis-*like, spherical, thin-shelled, colourless. Usually passed in proglottid, bound in packets containing ~10–20 eggs.	Most species 34–60 × 20–45, *R. siriraji* larger (100–115 × 38–42 *μ*m); Oval, colourless shell with visible vitelline membranes; embryophore tapered to spindle-shaped around oncospheres, Usually passed in proglottid, contained in polygonal capsule with 1–4 eggs (for known zoonotic spp.).	35 × 50 *μ*m in diameter. Usually passed in proglottid, contained in polygonal capsule with 8–15 eggs.	40 × 30 *μ*m; Slightly oblong to tapered, colourless, lacking a shell but with a thin embryophore. Passed contained in intact proglottid; dies quickly when released from parauterine organ.
Immature stage^e^	Cysticercoid	Cysticercoid	Cysticercoid	Likely cysticercoid	Tetrathyridium

a Disputed; see relevant section(s) in text.

b Not a valid name, but frequently reported in historical literature; most likely represents a combination of *R. celebensis*, other *Raillietina* spp. and *I. madagascarensis.*

c Most *Raillietina* spp. infect avian definitive hosts, though these are not recognized as zoonotic species.

d Measurements and descriptions for gravid, posterior proglottids.

e Immature stage infective to the definitive host.

### Bertiella

#### Generic taxonomy and morphology

*Bertiella* species are cestodes of primates in both the New and Old Worlds; they also infect rodents, dermopterans and Australian marsupials. This is the only genus of the Anoplocephalidae tapeworms known to infect humans. Blanchard first described the genus as ‘*Bertia*’ in great apes. Stiles and Hassall (1902) revised the generic name to *Bertiella*, as the name ‘*Bertia*’ had already been applied to a group of terrestrial land snails (type strain *Bertia cambojiensis*) in 1888 by Ancey.

**Fig. 2.**
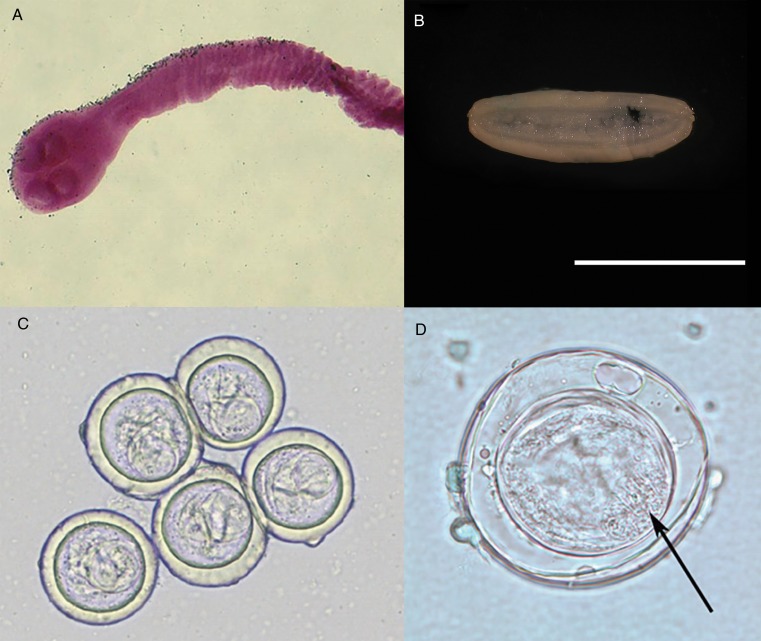
Specimens of *Bertiella studeri.* (A) Carmine-stained scolex; (B) single elongate, gravid proglottid (scale bar = 1 cm), (C) multiple eggs showing pyriform apparati; (D) singular egg, showing oncosphere with hooklets (arrow). Photos courtesy of DPDx, Centers for Disease Control and Prevention.

*Bertiella* spp. scolices are sub-globose with a rudimentary, unarmed rostellum (Fig. 2A) (Belding, 1965). The suckers are large and round; two on the ventral surface and two on the dorsal surface (Schmidt, 1986). The base of the scolex is markedly differentiated from the neck of the worm. Proglottids are craspedote, extended transversely and are far wider than they are long (Belding, 1965; Schmidt, 1986). The longitudinal osmoregulatory canals (excretory ducts) are paired dorsally and ventrally on either side of the proglottid, the ventral duct being much larger than the dorsal duct (Stunkard, 1940; Stunkard *et al*., 1964). A large coiled transverse canal connects the longitudinal canals (Stunkard, 1940; Beveridge, 1985; Foitová *et al*., 2011). Mature proglottids possess a single genital pore; this single genital opening irregularly alternates left to right across the length of the strobila (Foitová *et al*., 2011). The central ovary is fan-shaped and situated on the poral side of the midline (Stunkard *et al*., 1964), and the ‘C’-shaped vitellarium is dorsal and posterior to the ovary (Stunkard *et al*., 1964; Beveridge, 1985). A single wide transverse uterus across the centre of the proglottid may extend past the longitudinal osmoregulatory canals. Testes are anterior and dorsal, forming a transverse band which, depending on the species, may or may not extend beyond the longitudinal osmoregulatory canals (Stunkard, 1940; Stunkard *et al*., 1964; Beveridge, 1985; Galán-Puchades *et al*., 2000; Foitová *et al*., 2011). As the proglottids become gravid, the uterus swells with eggs to fill the entirety of the proglottid (Fig. 2B) and the uterus branches into elongate posterior and anterior diverticula as it expands (Beveridge, 1985; Galán-Puchades *et al*., 2000). The vitellarium, testes and ovary become diminished, but the cirrus sac, vagina and seminal receptacle are retained (Stunkard *et al*., 1964).

Ovoid eggs (Fig. 2C) are liberated from the proglottids and shed into host feces (Belding, 1965). These mature eggs have a shell and an inner envelope with an albuminous layer between. The delicate inner envelope contains a distinctive pyriform apparatus (Galán-Puchades *et al*., 2000). A bicornate protrusion at the apex of the pyriform apparatus is made up of two opposed tubular components and is only visible when viewed along the longitudinal axis of the pyriform apparatus (Beveridge, 1985). Distinct filaments extend out from the two arms of the bicornate protrusion (Beveridge, 1985). Within the pyriform apparatus, a distinctive, round, active oncosphere containing hooklets may be observed (Stunkard, 1940; Beveridge, 1985) (Fig. 2D). When observed in hyperosmotic solutions, such as seen during salt flotation, the eggs may appear flattened on one side or irregular in form, with folds, wrinkles or even vacuolated regions observed in the shell (Stunkard, 1940).

#### Life cycle and hosts

Currently, there are 29 known species of *Bertiella* infecting Marsupialia, Rodentia and Dermoptera in Asia, Papua New Guinea and Australia (Beveridge, 1985; Denegri and Perez-Serrano, 1997) and primates from Asia, Africa, South America and some Caribbean and Indian Ocean islands (Denegri and Perez-Serrano, 1997). The adult tapeworms reside within the lower two-thirds of the small intestine of the definitive host, attached by the four suckers present on the scolex (Belding, 1965). In addition to liberated eggs, mature gravid proglottids may also be passed in the feces individually or in strobilar fragments of around 8–16 segments. Based on the activity of the oncosphere, eggs are likely infective to the intermediate host immediately after passage. In dry conditions, oncospheric embryos gradually reduce the activity and die after approximately 1 week. Hot temperatures may also inactivate the eggs. When stored in cool, moist environments, oncospheres remained active after 2–3 months. When stored in water, most died after 5–6 weeks (Stunkard, 1940).

Oribatid mites are the intermediate hosts of *Bertiella* species and many other Anoplocephalidae (Fig. 1) (Denegri, 1993). These mites consume *Bertiella* eggs in the environment and oncospheres will hatch within the mite. Cysticercoids begin to form within 9 days; they are pyriform in shape and measure 130–160 × 100–120 *μ*m with a visible invaginated scolex (Stunkard, 1940). In one experiment, infected mites dissected 76 days after exposure contained identifiable cysticercoids, though it could not be determined if these were still viable (Stunkard, 1940). These intermediate host mites live naturally in cool and moist soil and frequently fruit (Stunkard, 1940; Denegri and Perez-Serrano, 1997). Consumption of vegetation, fruit or soil containing mites by primate definitive hosts completes the life cycle (Fig. 1).

#### Zoonotic species

Only *Bertiella studeri* and *B. mucronata* are currently recognized as infecting humans (Denegri and Perez-Serrano, 1997), though *B. studeri* may in fact represent a species complex that may include some zoonotic members. Following the resurrection of *B. satyri* and its separation from *B. studeri* (Foitová *et al*., 2011), early reports of *B. satyri* infection of humans may warrant re-investigation (Chandler, 1925). Regardless of the species, primates are the reservoir hosts of all *Bertiella* species currently recognized as infecting humans.

*Bertiella studeri:* Blanchard's original description of *Bertiella* contained two separate species of the new genus of cestode, including *Bertia* [sic] *satyri* in a Bornean orangutan (*Simia satyrus*; now *Pongo pygmaeus*) and *Bertia* [sic] *studeri* from an African chimpanzee (*Troglodytes niger*; now *Pan troglodytes*). In 1927, Baer synonymized *B. studeri* and *B. satyri*, with *B. studeri* as the senior synonym (Baer, 1927). However, recent work based on a molecular and morphologic investigation by Foitova *et al*. has resurrected the species *B. satyri* (Foitová *et al*., 2011). This report also suggested that many Old World *Bertiella* human infections from outside of Africa reported as *B. studeri* may represent *B. satyri* or another species of *Bertiella* (Foitová *et al*., 2011). Furthermore, investigation of multiple *B. studeri*-type specimens by Galán-Puchades *et al*. (2000), expanding on earlier observations by Spasski (1951) suggested that *B. studeri* should be considered a species complex containing at least four separate types; *B. studeri* sensu (Stunkard, 1940), *B. studeri* sensu (Bourquin, 1905), *B. studeri* sensu (Kagei *et al*., 1992) and *B. studeri* sensu (Ando *et al*., 1996).

Reported *B. studeri* morphology and morphometrics may be compromised by the possibility raised by Galán-Puchades *et al*. that this cestode in fact represents a species complex. Further investigation of whether the differences in morphology indeed do represent different species or sub-species, or simply reflect natural variation between different individuals or hosts, is warranted. The difficulties in determining the true number of species comprising ‘*B. studeri*’ has indicated the urgent need for comparative genotyping of specimens from different geographic locations and hosts, with particular references to primates, including humans, to elucidate the true nature of species within the genus.

*Bertiella studeri* measure up to 300 mm long by up to 15 mm wide at the widest proglottid and up to 2.5 mm thick (Stunkard, 1940). Mature to post-mature gravid proglottids are 7.8–11.3 mm wide (mean 9.52 mm) by 1.43–2.55 mm long (mean 1.76 mm) (Stunkard, 1940). The adult worm may contain up to 600 proglottids in a single chain. Scolices measurements have been between 475 and 800 *μ*m in diameter with the four oval suckers between 220 and 345 *μ*m in diameter at the widest point and about 200 *μ*m deep (Stunkard, 1940; Stunkard *et al*., 1964; Bhagwant, 2004). The neck is approximately 2.65–5.0 mm long (Stunkard, 1940). The proglottids increase in length and width with age. Stunkard (1940; Stunkard *et al*., 1964) described the development of the organs in a *B. studeri* taken from a rhesus monkey (*Macaca mulatta*) in captivity and another from a human child, with the development of genital organs starting between 90^th^ and 130^th^ segment distal to the scolex. Between segments 150 and 265, the genital organs appeared fully mature, with the uterus starting to fill with eggs by segments 330–350, and gravid proglottids filled with eggs were seen by segment 366 (Stunkard, 1940). Mature proglottids contained approximately 225–280 testes which may be between 36 and 95 *μ*m in diameter (Stunkard, 1940; Denegri and Perez-Serrano, 1997; Galán-Puchades *et al*., 2000) but averaged 66 × 83 *μ*m in one specimen (Galán-Puchades *et al*., 2000). The testes do not extend beyond the longitudinal osmoregulatory canals (Stunkard, 1940; Galán-Puchades *et al*., 2000) but the enlarged gravid uterus sometimes does. The unarmed cirrus sac measured between 360 and 440 *μ*m in length and 700 and 1100 *μ*m in width in one specimen (Stunkard *et al*., 1964) and 280–480 × 80–100 in another (Galán-Puchades *et al*., 2000). The ovary is positioned on the poral side of the midline and has been variously measured in three specimens as between 0.80 and 1.50 mm in diameter (Stunkard, 1940; Stunkard *et al*., 1964; Bhagwant, 2004). The C-shaped vitellarium is antero-lateral and opens towards the poral side and 500–600 *μ*m wide in one specimen (Stunkard *et al*., 1964), 230–360 in another (Galán-Puchades *et al*., 2000). The dorsally situated shell gland is 180–220 *μ*m in diameter. The seminal receptacle measures between 200 and 300 *μ*m long in one specimen (Stunkard *et al*., 1964) and 340–480 in another (Galán-Puchades *et al*., 2000). The vagina is 51–110 *μ*m long and 70–90 *μ*m wide (Galán-Puchades *et al*., 2000).

The eggs of *B. studeri* are oval to irregularly ovoid, measuring 33–46 *μ*m in width by 36–65 *μ*m in length (Stunkard, 1940; Stunkard *et al*., 1964; Beveridge, 1985; Ando *et al*., 1994; Denegri and Perez-Serrano, 1997; Galán-Puchades *et al*., 2000; Bhagwant, 2004; Sun *et al*., 2006; Sharma *et al*., 2017). Notable size variance has been observed between hosts, which may represent natural variation, or may indicate multiple species within a *B. studeri* complex. For example, eggs passed by a human, probably infected in Kenya, were 37–51 *μ*m (mean 45 *μ*m) by 37–46 *μ*m (mean 44 *μ*m). The pyriform apparati within these eggs measured 19–28 *μ*m in diameter (mean 23 *μ*m) and the oncospheres in this specimen measured between 12 and 15 *μ*m in diameter (mean 13 *μ*m) (Galán-Puchades *et al*., 2000). This contrasts with larger eggs recovered from a captive *M. mulatta* which were 40–60 *μ*m in diameter, containing pyriform apparati measuring 27–35 *μ*m by 20–21 *μ*m and oncospheres measuring 17–18 *μ*m by 19–20 *μ*m (Stunkard, 1940). It should be noted that the morphological features provided here for *B. studeri* are based on several samples taken from human infections. Recent work on the phylogenetic relationships of *Bertiella* species (Doležalová *et al*., 2015) has suggested that multiple species may infect humans in the Old World and thus some of the data summarized here may have been found to include multiple species on which molecular identification had been performed.

Reports exist from a wide variety of Old World primate hosts, including humans (*Homo sapiens*), chimpanzees (*P. troglodytes*), baboons (*Papio ursinus* and *Papio doguera*) and various monkeys (*Chlorocebus sabaeus*, *M. mulatta*, *Macaca cynomolgus*, *Macaca fascicularis*, *Macaca fuscata*, *Macaca radiata*, *Macaca syrichta syrichta*, *Macaca syrichta fascicularis*, *Cercopithecus aethiops pygerythrus*, *Cercopithecus nictitians schmidtii*, *Cercopithecus neglectus*, *Cercopithecus mona mona*, *Cercopithecus sabaensis*, *Cercopithecus aethiops cyanosusus*, *Cercopithecus sinicus*, *Cercopithecus fascicularis*) and gibbons (*Hyalobates hoolock*) (Ando *et al*., 1994; Denegri and Perez-Serrano, 1997). As noted previously, reports in Bornean orangutans (*P. pygmaeus*) may represent another species, *B. satyri* (Chandler, 1925; Foitová *et al*., 2011). Identified intermediate host oribatid mites of *B. studeri* are *Schlerobates laevigatus* and *Galumna* species (Stunkard, 1940) as well as *Scutoverix minutus* and *Archipeteria coleoptrata*. One report of *B. studeri* in a domestic dog from the Philippines is unexpected and may represent the transient passage of eggs following coprophagy (Africa and Garcia, 1935).

The first human case of *B. studeri* infection was reported (as *B. satyri*) from a child in Mauritius by Blanchard (1913). To date, 83 human cases of *B. studeri* infection have been reported, most acquired in Africa, Mauritius, the Middle East or South East Asia (Table 2). Several cases reported from countries where monkeys are not naturally found have included a history of contact with pet monkeys or zoological gardens (Denegri and Perez-Serrano, 1997). Infections are predominantly seen in children, though adults may also be affected (Denegri and Perez-Serrano, 1997). Humans are accidental hosts and human bertiellosis is most often seen in those with a history of association with monkeys or other non-human primates (Paçô *et al*., 2003). Some reported eating fruit in areas inhabited by monkeys (Bhagwant, 2004). Most infections have been reported in children (Denegri and Perez-Serrano, 1997). Adult *B. studeri* infecting humans have a lifespan of at least 2 years (Thompson *et al*., 1967). *Bertiella studeri* is generally considered to be restricted to the Old World, except for the Caribbean island of St. Kitts where one human case occurred and was traced back to the local green monkeys (*C. sabaeus*) which are of West African origin (Cameron, 1929). One puzzling case report of human infection with *B. studeri* from Brazil has been reported, based on the morphology of the proglottids and eggs. However, the morphometric data reported for these features are also consistent with *B. mucronata* (Lopes *et al*., 2015). This case may represent a misidentification of *B. mucronata* as *B. studeri*, but as discussed later in this review, the true taxonomic distinction and geographic distribution of these two species are not entirely clear and infections identified as ‘*B. studeri*’ may represent multiple species. Bertiellosis is traditionally considered to be an innocuous infection in human hosts (Denegri and Perez-Serrano, 1997). However, in several recent cases, various symptoms including abdominal distension, dyspepsia, nausea, diarrhoea, anorexia and perianal itching have been reported (Bhagwant, 2004; Sun *et al*., 2006; Sharma *et al*., 2017). Furthermore, the passage of strobilae per rectum either spontaneously or after treatment may cause a psychological disturbance in some patients.

**Table 2.** Country of acquisition, age of patient and egg sizes reported for all published human cases of bertiellosis to date

Geographical location of acquisition	Age (years)	Reported egg size (*μ*m)	Source	Notes and comments
*Bertiella studeri*				
Mauritius	8	nd	(Blanchard, 1913)	
India (Bihar or West Bengal)	2	46–49	(Chandler, 1925)	Originally reported as ‘*B. satyri*’*
India (West Bengal)	nd	nd	(Mukerji, 1927)	Originally reported as ‘*B. satyri*’*
West Indies (St. Kitts)	‘young’	nd	(Cameron, 1929)	St Kitts has monkeys of west African origin
India (Meghalaya)	nd	nd	(Sharma, 1930)	Originally reported as ‘*B. satyri*’*
India (Meghalaya)	nd	nd	“	Originally reported as ‘*B. satyri*’*
India (Meghalaya)	nd	nd	“	Originally reported as ‘*B. satyri*’*
India (East Bengal)	8	46–49	(Maplestone, 1930)	
Indonesia (Sumatra)	nd	nd	(Joyeux and Dollfus, 1931)	
Mauritius	8	nd	(Adams and Webb, 1933)	
Mauritius	4	nd	“	
Mauritius	7	nd	(Adams, 1935)	
Philippines (Iloilo)	8	55–73	(Africa and Garcia, 1935)	
India (Uttar Pradesh)	5	nd	(Maplestone and Riddle, 1936)	
Bangladesh	8	nd	(Roy, 1938)	
Indonesia (Java)	7	nd	(Bonne, 1940)	
Kenya	8	nd	(Buckley and Fairley, 1950)	
Indonesia (Java)	4	nd	Lie Kian, 1961 (Pers Comm)	
Indonesia (Kalimantan)	3.5	nd	“	
Singapore	6	nd	(Desowitz *et al*., 1961)	
USA (Minnesota)	5	40–60	(Stunkard *et al*., 1964)	Patient had close contact with a pet monkey
Yemen	nd	nd	(Fogh and Seaton, 1967)	
Yemen	6	nd	(Thompson *et al*., 1967)	Diagnosed in Britain in a returned traveller, reported as ‘*Bertiella* sp.’*
Indonesia (Sumatra)	6.5	nd	(Kwo and Koh, 1968)	
Indonesia (Sumatra)	7	nd	“	
Indonesia (Sumatra)	6	nd	“	
Indonesia (Sumatra)	14	nd	“	
Indonesia (Sumatra)	5	nd	“	
Indonesia (Sumatra)	6	nd	“	
Democratic Republic of Congo	‘young’	nd	“	
Republic of Congo	2.5	45–47 × 48–50	(Jones *et al*., 1971)	Diagnosed in Canada in a returned expatriate, reported as ‘Bertiella sp.’*
Sri Lanka	‘pre-school child’	nd	(Edirisinghe and Cumararajan, 1976)	Reported based on pers. comm. from Wijesundera, M.K. de S. 1975
Sri Lanka	‘pre-school child’	nd	“	Reported based on pers. comm. from Wijesundera, M.K. de S. 1975
Sri Lanka	4.5	nd	“	
Yemen	25	nd	(Imamkuliev *et al*., 1983)	Diagnosed in Russia from a Yemeni student
India	29	nd	(Subbannayya *et al*., 1984)	Reported as ‘Bertiella sp.’*
Thailand	26	nd	(Bhaibulaya, 1985)	
Saudi Arabia	28	nd	(Bolbol, 1985)	Reported as ‘Bertiella sp.’*
Gabon	2	nd	(Richard-Lenoble *et al*., 1986)	
India (West Bengal)	9	44 × 48	(Bandyopadhyay and Manna, 1987)	
Sri Lanka	‘child’	nd	(Weerasooriya *et al*., 1988)	
Sri Lanka	‘child’	nd	‘	
Indonesia (Sumatra)	8	nd	(Kosin and Kosin, 1992)	
Indonesia (Kalimantan)	5	nd	“	
Indonesia (Sumatra)	3.5	nd	“	
Indonesia (Sumatra)	‘child’	nd	“	
Indonesia (Sumatra)	‘child’	nd	“	
Indonesia (Sumatra)	‘child’	nd	“	
Indonesia (Sumatra)	‘child’	nd	“	
Indonesia (Sumatra)	‘child’	nd	“	
Indonesia (Sumatra)	3	nd	(Kagei *et al*., 1992)	
Indonesia (Sumatra)	‘adult’	nd	“	
Japan	3	nd	(Kojima *et al*., 1992)	
Japan	2	nd	(Iseki *et al*., 1993)	
India (Orissa)	4	48–62	(Panda and Panda, 1994)	
Kenya	33	37–51 × 37–46	(Galan-Puchades *et al*., 1995)	Diagnosed in Spain from a returned traveller
Japan	23	47–54	(Ando *et al*., 1996)	
Equatorial Guinea	50	51–49	(Galán-Puchades *et al*., 1997)	
Sri Lanka	10.5	nd	(Karunaweera *et al*., 2001)	
Vietnam	4	41–50	(Xuan le *et al*., 2003)	
Sri Lanka	2.5	nd	(Gallella *et al*., 2011)	
Saudi Arabia	40	nd	(El-Dib *et al*., 2004)	
Mauritius	3–7	50–65 × 30–45	(Bhagwant, 2004)	
Mauritius	3–7	nd	“	
Mauritius	3–7	nd	“	
Mauritius	3.5	nd	“	
Mauritius	32	nd	“	
South Africa (Western Cape)	28	nd	(Frean and Dini, 2004)	Probably acquired at a private zoo in Cape Town
Sri Lanka	2.5	nd	(Morawakkorala *et al*., 2006)	
Sri Lanka	5	nd	“	
China (Anhui)	3.5	38–50	(Sun *et al*., 2006)	
Yemen	‘Man’	nd	(Achir *et al*., 2008)	Diagnosed in Algeria from a Yemeni student
‘Africa’	‘young’	52–61	(CDC-DPDx, 2009)	Diagnosed from a refugee living in Australia
Saudi Arabia (Jizan)	adult	49–51	(Al-Mathal *et al*., 2010)	Diagnosed in Egypt from a recently returned expatriate
Equatorial Guinea	4	49–60	(Lozano Mdel *et al*., 2010)	
Indonesia (Sumatra)	2.5	nd	(Anwar and Ghiffari, 2010)	
India (Haryana)	4	44–36	(Malik *et al*., 2013)	
South Africa (Gauteng)	6	nd	(Du Plooy, 2014)	
South Africa (Gauteng)	27	nd	“	
India (Himachal Pradesh)	2	46–65	(Sharma *et al*., 2017)	
*Bertiella mucronata*				
Cuba	‘young’	36–40	(Cram, 1928)	
Brazil	29	nd	(Pessoa, 1930)	
Argentina	46	nd	(Bacigalupo, 1949)	
Paraguay	29	38–46	(d' Alessandro *et al*., 1963)	
Brazil (Minas Gerais)	nd	38–41	(de Costa *et al*., 1967)	
Argentina	45	nd	(Feldman *et al*., 1983)	
Argentina	2	nd	(Garaguso and Mendez, 1983)	
Brazil (Goiás)	2	39–41	(Paçô *et al*., 2003)	
Argentina (Corrientes)	9	nd	(Gené *et al*., 2011)	
Brazil (Pará state)	4	42–47 × 41–46	(Furtado *et al*., 2012)	
Brazil (Minas Gerais)	8	40 × 41	(da Silva *et al*., 2011)	
Brazil (Minas Gerais)	2.5	42–47	(Lopes *et al*., 2015)	Reported as ‘*B. studeri*’*

*Note the species categories presented in this table are based on geography (*B. studeri* from the Old World, *B. mucronata* from the New World).

The diagnosis of *B. studeri* infection may be undertaken by the demonstration of distinctive eggs and/or proglottids of the appropriate size and morphology in a patient's stool. These proglottids are active and motile; upon passage and storage overnight at 4°C, they may induce relaxation to allow microscopic examination (Malik *et al*., 2013). Clearing whole proglottids and staining these with carmine or a similar dye may assist in the detection of identifying internal structures. Travel history should be carefully taken and include any contact with primates from areas where *B. studeri* is endemic. The recovery of the scolex following treatment may assist in the confirmation of the diagnosis if possible but may not be possible (Denegri and Perez-Serrano, 1997). To definitively confirm species identity and to assist in determining the true nature of *B. studeri* as a species, collection of proglottids into 70% ethanol or another DNA-supportive fixative (not formalin), followed by submission to a reference laboratory for further molecular identification, is strongly indicated.

Praziquantel and niclosamide have been used successfully for the treatment of several recent cases (Karunaweera *et al*., 2001; Bhagwant, 2004; Sun *et al*., 2006; Al-Mathal *et al*., 2010; Gallella *et al*., 2011; Malik *et al*., 2013). However, apparent treatment failure with niclosamide occurred in some recent reports (Al-Mathal *et al*., 2010; Gallella *et al*., 2011). In one case, subsequent to niclosamide failure, the patient was successfully treated with 6 days of extract of Myrrh (*Commiphora molmol*) herbal therapy (Al-Mathal *et al*., 2010) rather than the conventional praziquantel therapy.

*Bertiella mucronata*: In 1895, Meyner identified a New World monkey tapeworm, which was initially described from two samples collected by Neumeister in 1888 from black howler monkeys (*Mycetes niger*, now *Alouatta caraya*) in Paraguay. At the time, Meyner considered the genus ‘*Bertia*’ (now *Bertiella*) to represent only a sub-genus of *Taenia* and thus this parasite was called ‘*Taenia (Bertia) mucronata*’ (Meyner, 1895). Stiles corrected this name to ‘*Bertia mucronata*’ in 1897 and later (with Hassall) (Stiles and Hassall, 1902) revised this genus name to ‘*Bertiella*’ due to the detected homonymy.

The morphometrics of all reported human cases are summarized, with a comparative table of variations between reported ‘*B. studeri*’ and *B. mucronata*, in Table 3. Notable differences between *B. mucronata* and *B. studeri* are their respective geographic distributions, a lower number of testes, a smaller cirrus sac diameter and a longer vagina in *B. mucronata*. Few complete *B. mucronata* individual specimens have been measured, one from a titi monkey (Callicebinae) in Peru was 256 mm long (Gomez-Puerta *et al*., 2009), while an infected human in Paraguay passed strobili up to 250 mm in length (d’ Alessandro *et al*., 1963). The longest complete strobila from one probable *B. mucronata* from a chimpanzee at a zoo in Cuba (although the primate was originally from Africa, it was considered that the cestode was locally acquired) measured 400 mm (Cram, 1928). Width of the widest mature proglottid has been measured as 7.5–10 mm wide (Cram, 1928; d’ Alessandro *et al*., 1963; de Costa *et al*., 1967; Gomez-Puerta *et al*., 2009), gravid proglottids may measure up to 14 mm wide by 3 mm in thick (Cram, 1928). One mature individual worm from a human in Brazil possessed 700 proglottids (de Costa *et al*., 1967). The scolex measured 860 *μ*m in diameter in one individual (de Costa *et al*., 1967) and 340 × 450 *μ*m in another (Gomez-Puerta *et al*., 2009). The four oval suckers have been reported as either 240 × 260 *μ*m (de Costa *et al*., 1967) or 210 *μ*m (Gomez-Puerta *et al*., 2009) in diameter at the widest point. The neck is approximately 200–300 *μ*m long (Cram, 1928). Proglottids contain up to between 120 and 140 testicular follicles, which are variously reported as 38–41 *μ*m (de Costa *et al*., 1967), 60 *μ*m (d’ Alessandro *et al*., 1963) and 80–100 *μ*m (Gomez-Puerta *et al*., 2009) in diameter. The seminal receptacles measured 265 × 130 *μ*m in one specimen (d’ Alessandro *et al*., 1963), but may grow up to a dimeter of between 350 and 400 *μ*m in mature proglottids (d’ Alessandro *et al*., 1963; de Costa *et al*., 1967). The unlobed ovary is located in the centre of the proglottid and is 1.3 × 1.7 mm in diameter (de Costa *et al*., 1967). The cirrus sac averages 267 *μ*m in length (d’ Alessandro *et al*., 1963). In mature proglottids, the vagina is 1.03–1.60 mm long, but may reach 2 mm in width when fully gravid (de Costa *et al*., 1967). Reported egg sizes from human cases of *B. mucronata* vary between 36 and 47 *μ*m in maximum diameter (Stiles and Hassall, 1902; Cram, 1928; Furtado *et al*., 2012). Although eggs of *B. mucronata* are on average smaller than *B. studeri*, there is a considerable degree of overlap (Cram, 1928; d’ Alessandro *et al*., 1963; de Costa *et al*., 1967; Paçô *et al*., 2003; Gomez-Puerta *et al*., 2009; da Silva *et al*., 2011; Lopes *et al*., 2015). The pyriform apparatus within these eggs has been measured as 21–45 *μ*m in diameter at the widest point and oncospheres as 9–16 *μ*m in diameter (d’ Alessandro *et al*., 1963; Gomez-Puerta *et al*., 2009; Furtado *et al*., 2012; Lopes *et al*., 2015).

**Table 3.** Comparative morphometrics of *Bertiella studeri*, *Bertiella mucronata* and *Bertiella satyri* proglottids from selected references

	*B. studeri*				*B. mucronata*			*B. satyri*	
	Bhagwant (2004)	Stunkard (1940)	Stunkard, *et al*. (1964)	Galan-Puchades *et al*. (2000)	d'Alessandro *et al*. (1963)	de Costa, *et al*. (1967)	Cram (1928)	Chandler (1925)	Foitova *et al*. (2011)
	*Homo sapiens*	*Homo sapiens*	*Homo sapiens*	*Homo sapiens*	*Homo sapiens*	*Homo sapiens*	*Homo sapiens*	*Homo sapiens*	*Pongo abelli*
Gravid proglottid width (mm)	9.98	15	6	7.8–11.3	nd	14	14	11	14
Gravid proglottid length (mm)	nd	1	6.0	1.43–2.55	nd	nd	nd	1	2.9
Gravid proglottid thickness (mm)	1.82	3	1.76	nd	nd	nd	3	1.5	nd
Number of testes	214–225	‘about’ 250	nd	280	120–140	132	‘about’ 100	75	116–124
Diameter of testes (*μ*m)	76–69	36–95	70–100	68–80	60	38–41	80–100	65–70	52–111
Maximum diameter of cirrus sac (*μ*m)	600–900	300–500	360–440	280–480	267	nd	nd	310	322
Maximum diameter of ovary (mm)	1.15–0.38	0.5–0.9	0.80–1.5	1.54–0.37	nd	1.7	nd	nd	nd
Maximum length of seminal receptacle (*μ*m)	360–900	200–300	200–300	480–670	265	400	nd	360	nd
Maximum width of seminal receptacle (*μ*m)	200–450	nd	nd	310–480	130	240	nd	nd	nd
Maximum length of vagina (*μ*m)	330–540	400–600	nd	1100–1170	nd	1600	nd	>500	790–984
Alternation of genital pores	irregular	nd	nd	irregular	nd	nd	irregular	nd	irregular

New World monkeys are the natural definitive hosts of *B. mucronata*. Reported species found to be infected are *A. caraya*, *Alouatta guariba clamitans*, *Callicebus personatus nigrifrons*, *Callicebus oenanthe*, *Cebus apella fatuellus*, *Cebus capucinus* and *Callithrix sagui* (Denegri and Perez-Serrano, 1997; de Souza Júnior *et al*., 2008; Gomez-Puerta *et al*., 2009). Recognized intermediate hosts of *B. mucronata* are *Dometorina suramericana* and *Schlerobates atahualpensis* (Denegri, 1993).

Eleven human cases of *B. mucronata* infection have been reported in the literature thus far. As with *B. studeri*, the majority were in children, but four of the infections were reported from adults. All cases have originated in South America (Brazil, Northern Argentina and Paraguay) or Cuba (Table 2) with over half of these reported from Brazil. Non-human primate infections have been reported from Peru (Gomez-Puerta *et al*., 2009). Many historical case reports did not include clinical history, but one case from Paraguay reported intermittent constipation and diarrhoea (d’ Alessandro *et al*., 1963), while another from Brazil reported only nocturnal abdominal distension (Furtado *et al*., 2012). Two cases from Brazil presented with marked symptoms of abdominal pain, combined with intermittent vomiting, dyspepsia, anorexia and diarrhoea (Paçô *et al*., 2003; da Silva *et al*., 2011). All cases with clinical history reported the passage of tapeworm proglottids and/or strobilae *per rectum* (d’ Alessandro *et al*., 1963; de Souza Júnior *et al*., 2008; da Silva *et al*., 2011; Furtado *et al*., 2012; Lopes *et al*., 2015). Recently reported cases of bertiellosis due to *B. mucronata* for which treatment is described have all been successfully treated with praziquantel (Furtado *et al*., 2012; Lopes *et al*., 2015).

The most recent *Bertiella* infection reported from Brazil (Lopes *et al*., 2015) was identified as *B. studeri* based on limited features of proglottid morphology, egg size and morphology. While human infections from South America have traditionally been identified as *B. mucronata* based on geography and egg size, there is a significant variation in the egg diameters reported from these infections (Table 3). Recent molecular work found that a ‘*B. mucronata*’ isolate from a human in Brazil most closely clustered with *B. studeri* and *B. satyri* specimens from non-human primates and a human in Africa and Asia. Based on these findings, it seems likely that multiple species of *Bertiella* species may infect humans in the New World and the Old World, and that traditional morphologic identification and species assignment to human infections will require future revision in consideration of molecular findings.

#### Differentiating features of currently reported human-infecting species

Although early reports of *B. satyri* infection of humans probably represent a misidentification of *B. studeri*, this species can infect large primates and it remains unclear if some past human infections with this species may have occurred. We include it in this section to allow the correct identification of any potential future *B. satyri* infections of humans. Comparative morphometrics of *Bertiella studeri*, *Bertiella mucronata* and *Bertiella satyri* proglottids are described in Table 3. *Bertiella mucronata* is found in the Americas only and has ⩽150 testes per proglottid, whereas as *B. studeri* has >200 (d’ Alessandro *et al*., 1963), *B. satyri* has thus far only reported from the Old World and has approximately 75–124 testes (Chandler, 1925; Foitová *et al*., 2011). The vagina is much longer in the examples of *B. mucronata* and *B. satyri*, extending medially from the edge of the proglottid to the ovary, well past the lateral osmoregulatory canals, whereas the *B. studeri* vagina does not extend past these canals for any considerable distance (Chandler, 1925; Cram, 1928; Stunkard, 1940; d’ Alessandro *et al*., 1963; Foitová *et al*., 2011). The cirrus sac is weakly developed and the vagina is strongly developed and muscular in *B. mucronata* and *B. satyri*, whereas in *B. studeri*, the cirrus sac is strongly developed and muscular, but a weakly developed, amuscular vagina is seen (Cram, 1928; Foitová *et al*., 2011). Chandler (1925) and Foitova *et al*. (2011) describe *B. satyri* as having regularly alternating genital pores whereas *B. studeri* does not, but the original description of *B. satyri* by Blanchard (1891; Blanchard, 1913) describes irregularly alternating genital pores. Furthermore, specimens of *B. studeri* well described by Galan-Puchades *et al*. (2000) and Bhagwant (2004) possess alternating genital pores. It should be noted that the former specimen had an unusually long vagina for *B. studeri* and may represent another, as yet unidentified, species of *Bertiella* (Galán-Puchades *et al*., 2000). The scolex of *B. satyri* is easily differentiated from that of *B. studeri* and *B. mucronata* by the presence of a knob-like projection (possibly a rudimentary rostellum) on its apex (Chandler, 1925; Foitová *et al*., 2011).

As noted previously, the taxonomy of *Bertiella* species was in flux during the late 19^th^ and early 20^th^ centuries. Five separate species names were synonymized by Baer (1927), into *B. studeri*, based on the opinion that variations in morphometrics in these five *Bertiella* species in Africa and Asia were not sufficiently pronounced to allow species differentiation. Simply, Old World isolates from humans were *B. studeri* and New World isolates were *B. mucronata* (except for those acquired on St. Kitts). Defined anatomical features and measurements may not be entirely reliable for species identification, as variations may be seen based on the stage of sexual maturity of the proglottids and on the degree of muscular contraction at the time of fixation (Stunkard, 1940). Furthermore, there is a wide variation in the reported size of the scolices and suckers and eggs, even within individual species. Finally, for both *B. studeri* and *B. mucronata*, very few individual specimens have thus far been thoroughly examined and documented to obtain reliable estimates of the size range of individual anatomical features. The recent resurrection of the human-infecting species *B. satyri* and the advent of molecular phylogenetics have made this simplistic approach to the taxonomic identification of human-infecting *Bertiella* species untenable.

#### Molecular biology

Relatively, few studies on the molecular biology of *Bertiella* spp. have been conducted. In one study, *B. studeri* specimens from Mauritius were determined to be monophyletic members of the family Anoplocephalidae based on 18s rRNA gene sequences. When these sequences were analysed by the distance-based neighbour-joining method, *Bertiella* and other Anoplocephalidae were found to be most closely related to the Hymenolepididae. However, this close relationship was not supported when the maximum parsimony method was employed (Taleb-Hossenkhan and Bhagwant, 2012).

A recent study employed more phylogenetic markers (*28S rRNA*, partial *5.8S-ITS2 rRNA*, *cox1* and *nad1*) to investigate the phylogeny of the genus *Bertiella* (Doležalová *et al*., 2015). This study compared the *Bertiella* sequences from multiple non-human primate and human sources in Africa, Asia and South America. *Bertiella* species from humans and non-human primates were found to be monophyletic and within the family Anoplocephalidae. However, analysis of 28s rRNA sequences found that the relationship of *Bertiella* from Australian rodents and marsupials was paraphyletic to other *Bertiella* species, with the authors suggesting that the latter might be split into a new genus. This work demonstrated a high degree of heterogeneity within the *Bertiella* spp. analysed. Sequences of the *nad1* locus showed close relationships between *Bertiella* species taken from *P. troglodytes* in Kenya, *H. sapiens* in Brazil, *Pongo abelii* in Indonesia and a human infection acquired in Equatorial Guinea. Analysis of the partial *5.8S-ITS2 rRNA* locus showed close phylogenetic relationships between the *Bertiella* sp. from a human in Brazil, *B. satyri* from *P. abelii* in Indonesia, *B. studeri* from *P. troglodytes* in Kenya, the Equatorial Guinea-acquired human *B. studeri* isolate and two ostensibly *B. studeri* isolates from *M. fuscata* in Japan. A separate group clustered a *B. mucronata* taken from *Callicebus oecanthe* in Peru with isolates from *P. troglodytes* in Uganda, *Gorilla gorilla* in the Central African Republic and an *Anoplocephala gorillae*-like cestode from a *Gorilla beringei* in Rwanda. The summary of this work is that humans and non-human primates may be infected with multiple species of *Bertiella* and that future species identification of infecting isolates should be performed using a combination of morphology and standardized molecular techniques.

### Dipylidium

#### Taxonomy and morphology

Known by many names such as the ‘dog tapeworm’, ‘double-pored tapeworm’, ‘flea tapeworm’ and ‘cucumber tapeworm’, *Dipylidium caninum* is a well-known parasite to pet owners and veterinarians. It is a member of the family Dipylidiidae, which includes various small- to medium-sized tapeworms that are parasitic in carnivorous mammals with retractable, typically armed rostella (Khalil *et al*., 1994; Hoberg *et al*., 1999). An important feature of the Dipylidiidae is that the gravid uterus is replaced by multiple egg capsules (Wardle and McLeod, 1952). Several species of *Dipylidium* have been described over the years, but many names are likely to be invalid. Venard (1937) regarded only three species of *Dipylidium* as valid; their occurrence in humans is not known and the two other non-*D. caninum* species are poorly studied. Thus, only *D. caninum* will be discussed here.

Grossly, *D. caninum* is a relatively robust tapeworm, measuring 10–70 cm in length and around 3 mm in width, comprised of about 60–175 proglottids. Immature proglottids are trapezoidal in shape, while gravid proglottids shed in feces have a crawling motility and a particular convex shape, often described as resembling cucumber seeds (thus leading to one of its many common names). *Dipylidium*, unlike many of the other more commonly-encountered human-infecting cyclophyllidea, has two genital pores per proglottid. The genital pores open approximately in the middle of the lateral margins and are arranged directly opposite to each other. The double-pored morphology may be apparent to the naked eye as small, subtle indentations on mature proglottids. There are two sets of reproductive organs within a proglottid, each associated with a genital pore. Bilobed ovaries and a web-like uterus are situated posterior to the opening, and a vas deferens and cirrus sac sit just anterior to the genital pore openings. The testes are numerous and occupy most of the space between the osmoregulatory canals. In fully mature terminal proglottids, the uterus breaks down into egg capsules that fill most of the proglottid. Eggs are present in clusters of usually 10–20 eggs bound by a thin membrane. Individual eggs are 40–50 *μ*m, colourless, spherical and with a thin shell and embryophore (Miyazaki, 1991).

The scolex is 0.25–0.50 mm wide and roughly spade-shaped when the muscular rostellum is extruded. The rostellum is armed with multiple transverse rows of ‘rose-thorn’ hooks. The number of rows is usually reported as three in *D. caninum*, but the actual number may be difficult to count due to a spiraled arrangement in some specimens (Venard, 1937).

#### Life cycle and hosts

Intact motile proglottids are shed in the feces of the infected definitive host or may actively crawl out of the anus. Egg capsules are released from the proglottid passively after motility ceases and the tegument dries and disintegrates. Larvae of the flea intermediate host ingest these eggs, and the oncospheres penetrate the digestive tract and enter the haemocoel. The oncosphere continues to undergo developmental changes as the larva pupates, and transformation to a cysticercoid is ultimately completed in the adult flea (about 30 days post infection). Only the larval stages of fleas are capable of ingesting the large egg packets, so infections cannot be initiated in adult fleas which have smaller mouthparts (López-Neyra and Muñoz, 1919; Venard, 1937). The common dog flea (*Ctenocephalides canis*) and the cat flea (*C. felis*) are the prototypical intermediate hosts, although the dog louse (*Trichodectes canis*) and human flea (*Pulex irritans*) may also reportedly act as intermediate hosts (López-Neyra and Muñoz, 1919; Miyazaki, 1991).

The canid and felid definitive hosts (and humans) become infected by the ingestion of adult fleas containing cysticercoids. The cysticercoid develops into an adult in the small intestine over about 20 days. Historical infection trials suggest that growth is slower in cats than dogs and that mature specimens derived from feline hosts appear more delicate (Venard, 1937). Newer molecular data suggest that genetic differences exist between cat and dog isolates, possibly explaining differences in parasite establishment and development (Beugnet *et al*., 2018). Apart from anal pruritus from proglottid migration, the natural hosts typically show no complications of infection, except in particularly heavy infections where gastrointestinal irritation can occur from the anchoring of the rostellum (Miyazaki, 1991).

*Dipylidium caninum* is truly a cosmopolitan parasite and is common in both wild and domestic canid and felid definitive hosts around the world – particularly where flea control is inadequate. However, prevalence is likely underestimated by many fecal flotation methods as egg clusters/proglottids can be too heavy to float and are not distributed evenly in the fecal sample (Blagburn, 2001).

#### Human infections

Numerous *D. caninum* infections in humans have been reported worldwide for over a century; however, it is still considered by most clinicians to be an unusual finding. The actual number of confirmed cases is difficult to ascertain as not all have been reported in literature; fewer than 100 case reports exist in English language literature, but within a 4-year span (1973–1977), the Centers for Disease Control received 43 requests for the contemporary drug of choice (niclosamide) in diagnosed cases (Molina *et al*., 2003). A recent search including Chinese language databases estimated that at least 349 human cases have been reported worldwide (Jiang *et al*., 2017). It seems a reasonable assumption that the true incidence of dipylidiasis in humans is underestimated, given the ubiquity of *D. caninum* in pet dogs and cats, and the fact that detailed examination of proglottids in clinical settings is seldom performed.

The vast majority of published infections have occurred in children under about 8 years, with a substantial proportion (estimated one-third) in infants <6 months old (Chappell *et al*., 1990; Cabello *et al*., 2011). A recent case series from one clinic reported a mean age of 3.8 years (7 months–10 years) among 10 cases diagnosed over a 2-year period (Portokalidou *et al*., 2018). Reports in adults are extremely uncommon. *Dipylidium caninum* was reported in an adult kidney transplant recipient presenting with frequent diarrhoea, but the individual eggs (i.e. not clusters) shown appear to represent *Hymenolepis* sp. and it is not stated whether proglottids were found (Sahin *et al*., 2015). A more convincing case exists from a 57-year-old in Australia who passed morphologically consistent proglottids and egg clusters (Jackson *et al*., 1977).

Transmission to humans occurs through the accidental ingestion of fleas from pet dogs or cats, or possibly by being licked if exposed cysticercoids happen to be present on the tongue of the pet after grooming. Unsurprisingly, many patients lived in or visited households with dogs. Some children were reported to have played ‘games’ with their dogs which could potentially lead to ingestion of fleas/cysticercoids (Turner, 1962; Molina *et al*., 2003; García-Agudo *et al*., 2014; Jiang *et al*., 2017). Close contact between children and dogs is often cited as a reason why the age pattern is observed, although close interaction with dogs is not uncommon among adults. For example, 56% of dog-owning adults report co-sleeping with their dogs (Chomel and Sun, 2011), and 50% allow face-licking (Overgaauw *et al*., 2009), suggesting that there may be other factors at play (e.g. age-related immunity) besides children's close interaction with pets. Contact with cats is less frequently described in dipylidiasis literature, but their potential contribution to environmental flea infestations and direct infection risk should not be ignored. Flea control strategies and/or infestation status of pets implicated in transmission are not usually reported, but occasionally pets are followed up and *D. caninum* infections confirmed (García-Agudo *et al*., 2014).

Typically, very few or no symptoms are reported (Reid *et al*., 1992; Taylor and Zitzmann, 2011; Portokalidou *et al*., 2018). Apart from psychological distress of both the child and parent from the passing of motile proglottids, symptoms such as diarrhoea, mild gastrointestinal pain, urticaria, poor appetite and anal pruritis have been reported (Samkari *et al*., 2008; García-Agudo *et al*., 2014). Infections are typically light, but occasionally >10 worms are found (Chappell *et al*., 1990). Note that evaluating the actual infection intensity may be difficult or impossible unless scolices are recovered and counted.

In several instances, proglottids are initially misidentified as fly larvae, rice grains or other helminths (Turner, 1962; Samkari *et al*., 2008; Szwaja *et al*., 2011). Infections have been mistaken for the nematode *Enterobius vermicularis* due to anal pruritis and misidentification of motile proglottids as female pinworms. Laboratory identification is generally straightforward based on the features of proglottids passed in feces (presence of two genital pores, egg clusters, overall size and shape); scolices are also of diagnostic value but are very seldom recovered (Samkari *et al*., 2008; Taylor and Zitzmann, 2011). Correct identification is important as some anthelmintics (e.g. benzimidazoles) used for treating pinworm infections have no activity against *D. caninum* (Roberson and Burke, 1982; Taylor and Zitzmann, 2011). As with nearly all intestinal cestodiases, praziquantel and niclosamide are the most widely employed treatments. Of note, apparent resistance to praziquantel has been observed in some dog infections, thus treatment of human cases should be carefully monitored (Chelladurai *et al*., 2018). Despite the growing concern over the efficacy of praziquantel against *D. caninum*, reports are still rare and research preliminary. Furthermore, many newer-generation, compounded ectoparasitic preventives (in both spot-on and collar formulations) have proven effective in preventing flea infestations and interrupting *D. caninum* transmission in pets (Fourie *et al*., 2012, 2013; Beugnet *et al*., 2017).

### Raillietina

#### Taxonomy and morphology

The genus *Raillietina*, one of many taxa named after the pre-eminent helminthologist Alcide Railliet, holds the distinction of being the largest genus of cestodes, comprised of nearly 300 species (Schmidt, 1986). It also represents the only group from the family Davaineidae (subfamily Davaineinae) known to infect humans. As such, this family is largely unknown to the medical parasitological community. The majority are parasites of birds, but some species are associated with mammalian hosts. The known human-infecting species appear to be parasites of rodents (*Raillietina celebensis*, *Raillietina demerariensis*, *Raillietina siriraji*). Davaineinids are characterized by the possession of a short, broad, retractable rostellum, typically armed with small T- or hammer-shaped hooks, and by small spines on the suckers. The subfamily Davaineinae includes members that bear egg capsules instead of uteri (Schmidt, 1986). Conventionally, *Raillietina* is further divided into several subgenera based on the number of eggs per capsule and the position of the genital pore (*Parioniella*: one egg/capsule, genital pores unilateral; *Skrjabinia*: one egg/capsule, genital pores irregularly alternating; *Raillietina*: multiple eggs/capsule, genital pores unilateral; *Fuhrmannetta*: multiple eggs/capsule, genital pores irregularly alternating) (Schmidt, 1986). Note that some authors treat these subgeneric divisions as full genera, though the straightforward, cleanly-delineated generic status of these groups does not appear supported by available molecular evidence (Khalil *et al*., 1994; Littlewood *et al*., 2008; de Oliveira Simões *et al*., 2017; Mariaux *et al*., 2017). Nevertheless, the *Raillietina* spp. described from human infections all belong to subgenus *Raillietina*, with unilateral genital pores opening in the anterior portion (usually the anterior quarter, sometimes the anterior third) of the lateral margin of the proglottid (Baer and Sandars, 1956). Specific determination is usually based on the characteristics such as the number of eggs/capsule, length of the cirrus pouch, number of testes, and the number and length of rostellar hooks.

Most *Raillietina* spp. relevant to zoonotic infections are about 10–51 cm in length and 2–3 mm wide. The scolex possesses a rostellum with numerous hammer-shaped hooks arranged in two circles (usually alternating large and small hooks) and sometimes a collar of minute spines directly behind the rostellum. Proglottids range in shape from broadly rectangular to nearly square with rounded corners further down the strobila, giving the posterior portion a beaded appearance. Egg capsules are apparent in more mature segments and have a polygonal shape (130–180 *μ*m in diameter) with a transparent, parenchymatous exterior zone and a dark, nearly opaque interior.

#### Life cycle and hosts

While the known zoonotic species are associated with rodent and not avian hosts, most of what is known about the development of *Raillietina* spp. is from experimental studies of poultry-infecting species (namely *Raillietina cesticillus*, *Raillietina echinobothrida* and *Raillietina tetragona*) (Horsfall, 1938; Reid *et al*., 1938). There is a lack of published experimental trials on mammal-associated *Raillietina* spp., so inferences based on avian-associated species should be considered tentative. It is not known whether avian *Raillietina* spp. are potentially zoonotic or even capable of infecting mammalian hosts (and *vice versa*). However, the majority of other zoonotic cestodes causing intestinal infections are associated with mammalian definitive hosts (e.g. *Dibothriocephalus*, *Adenocephalus*, *Spirometra*, *Dipylidium*, *Bertiella*, *Hymenolepis*, *Taenia*, etc.). The differences in digestive anatomy and physiology between mammalian and avian hosts perhaps do not permit the effective establishment of avian definitive host-adapted adult cestodes in humans.

With this caveat in mind, *Raillietina* spp. appear to follow a life cycle similar to *Bertiella* and *Dipylidium*, involving a single intermediate host and a cysticercoid stage (Fig. 1). Proglottids are shed in the feces of infected definitive hosts and are passed in a highly motile state. Migration to the outer surface of the feces has been observed, which seems to aid in its ultimate transmission to the arthropod intermediate host; however, no particular tropism or response to directional stimulus has been identified (Reid *et al*., 1938). Upon consumption, the oncosphere hatches from the egg and penetrates the intestinal wall of the arthropod. Cysticercoids develop free within the body cavity and are usually infectious after a period of 2–3 weeks. The scolex evaginates upon consumption by the intermediate host, likely in response to digestive enzymes. Early in infection (~5 days), most worms are recovered from the first quarter of the intestine, but in patent infections, the primary site of localization is in the second quarter of the intestine (Gray, 1972). It is not known whether this applies to mammalian hosts, but a similar pattern has been observed in rats infected with *Hymenolepis diminuta* (Gray, 1972). Prepatent periods are short in avian hosts; gravid *R. cesticillus* proglottids may be passed by chickens as soon as 13 days post-exposure (Reid *et al*., 1938). As knowledge of mammalian-infecting species is limited to field studies on naturally-infected hosts, nothing is known on their course of infection.

Numerous species have been identified as intermediate hosts for various *Raillietina* spp., primarily ants and beetles and occasionally terrestrial gastropods (Wardle and McLeod, 1952). Many beetle intermediate hosts are carnivorous or opportunistic species that actively prey upon motile proglottids, such as ground beetles (Carabidae), and also other beetles that feed on animal dung or detritus such as scarab beetles (Scarabaediae) and darkling beetles (Tenebrionidae) (Reid *et al*., 1938). The actual intermediate host specificity varies widely by *Raillietina* species, and is not known in many instances (Pradatsundarasar, 1972). In particular, this is poorly characterized for the rodent-associated zoonotic *Raillietina* spp., including the most commonly reported *R. celebensis* (Rougier *et al*., 1981). A preliminary report of zoonotic *R. siriraji* cysticercoids in cockroaches was eventually shown to be a misidentification of gregarine protozoa (Pradatsundarasar, 1972).

As stated previously, the majority of *Raillietina* species use avian definitive hosts, and the group includes well-known pathogens of poultry (Dunn, 1978). The zoonotic species have all been identified from various rodents, primarily the two cosmopolitan rat species *Rattus norvegicus* and *Rattus rattus* (Miyazaki, 1950; Chandler and Pradatsundarasar, 1957; Niphadkar and Rao, 1969; Tung *et al*., 2013). *Raillietina celebensis* has also been recorded from other native murid rodents, including Bandicoot rats (*Bandicota* spp.) of the Indian subcontinent and Southeast Asia, the Asian house shrew (*Suncus murinus*) and the trefoil-toothed giant rat (*Lenomys meyeri*) of Sulawesi (Baer and Sandars, 1956; Tung *et al*., 2009). The other Old World species *R. siriraji* has been identified in Polynesian rats (*Rattus exulan*s) and Asian house shrews along with the usual hosts (Chenchittikul *et al*., 1983; Roberts, 1991). Several surveys of rodents from endemic areas in Southeast Asia simply report *Raillietina* sp. from a variety of rodents which seem likely to represent either *R. celebensis* or *R. siriraji.* Among these, the chestnut white-bellied rat (*Niviventer fulvescens*), Edward's long-tailed giant rat (*Leopoldamys edwardsi*), fawn-coloured mouse (*Mus cervicolor*), red spiny rat (*Maxomys surifer*), white-toothed rats (*Berylmys* spp.) and other *Rattus* spp. (*R. losea*, *R. tanezumi*) represent potential hosts (Chaisiri *et al*., 2010, 2015; Herbreteau and Morand, 2011).

*Raillietina demerariensis* is more poorly understood, but reports under various synonyms exist from the New World rodents including the Cuban huita (*Capromys pilorides*) and hystrichomorph rodents (e.g. guinea pigs, capybara, etc.) (Cameron and Reesal, 1951; Baer and Sandars, 1956). The howler monkey *Alouatta seneculus* has also been described as a host for *R. demerariensis* and another species *R. alouattae*, although it is not known whether this represents the normal reservoir host or an aberrant host susceptible to infection (Baer and Sandars, 1956). Unidentified *Raillietina* sp. possibly representing *R. demerariensis* are reported from cosmopolitan *Rattus* spp. in Jamaica and Brazil (Waugh *et al*., 2006; Simões *et al*., 2016).

#### Human infections

*Identity of zoonotic species*: All reports of *Raillietina* sp. infections in humans appear to involve mammalian-associated species in the subgenus *Raillietina.* However, the precise identity and number of *Raillietina* species involved in human infections is a perplexing and unsettled subject on account of incomplete descriptions, fragmentary reference material, invalid and inconsistent naming, morphological variability and of course the enduring taxonomic ‘lumper/splitter’ dichotomy. The long, complex taxonomic history and debate are discussed at length by both Chandler and Pradatsundarasar (1957) and by Baer and Sandars (1956). In the absence of further modern characterization, the safest tentative conclusion appears to be that three valid species have recovered from humans: *R. celebensis* in the Old World, *R. demerariensis* from the New World and *R. siriraji* from Thailand. These names have an extensive list of synonyms and yet the validity of some species has not been definitively settled (Table 4).

**Table 4.** List of names generally regarded as synonyms for *Raillietina* spp. described from human infections

Name	Synonyms
*R. celebensis*	*Taenia madagascariensis*^a^ *Davainea madagascariensis*^a^ *Davainea celebensis* *Meggittia celebensis* *R. asiatica* *R. formosana* *R. funebris* *R. garrisoni* *R. murium* *R. madagascariensis*^a^ *R. sinensis* *R. siriraji*^b^
*R. demerariensis*	*Taenia demerariensis* *R. brumpti* *R. equatoriensis* *R. quitensis* *R. leoni* *R. luisaleoni* *R. halli*
*R. siriraji*	sp. nov. described by Chandler and Pradatsundarasar (1957)

(Baer, 1956; Baer & Sandars, 1956; Chandler & Pradatsundarasar, 1957; Fain *et al.*, 1977; Matevosyan, 1966).

a Name likely represents multiple Old World *Raillietina* spp. and *Inermicapsifer madagascariensis.*

b Regarded by Fain *et al*. (1977) as a synonym of *R. celebensis*, although treated as a valid species by others (Chandler and Pradatsundarasar, 1957; Matevosyan, 1966; Charoenlarp and Radomyos, 1973).

Of these species, *R. celebensis* has been most frequently described in human infections, although of course not always under that name (Table 4). Despite the fragmentary nature of descriptions, comparison across reported descriptions of *Raillietina* spp. from humans and sympatric rodents in the Old World reveals that differences in aspects such as overall dimensions, number of hooks, presence of rostellar spines, number of testes and the length of the cirrus pouch are trivial or too overlapping to warrant specific status (Joyeux and Baer, 1929; Baer and Sandars, 1956). Thus, treating such described species as one entity *R. celebensis* is probably appropriate. Further genetic studies will help to confirm or refute this.

Adding to the taxonomic upheaval, many historical reports across various countries have used the problematic name *R. madagascariensis*. The name is rightly regarded by Baer and Sandars (1956) as a *species sub judice* as it is impossible to determine the true identity of this name due to poor original type material and conflicting historical revisions (Joyeux and Baer, 1929; Baer, 1956). This was first highlighted during the examination of multiple *R. madagascariensis*-type specimens by Joyeux and Baer (1929), who reported poor states of preservation and misrepresentation; one such scolex was found to be an immature specimen of *Taenia saginata.* Furthermore, through the years, various workers have proposed revisions, not all of which were universally adopted. For example, Japanese authors synonymized multiple *Raillietina* spp. and even *R. demerariensis* of the New World into ‘*R. madagascariensis*’ (Miyazaki, 1950). However, it is worth nothing that Miyazaki later used *R. celebensis* in referring to the same works in his textbook nearly 40 years later (Miyazaki, 1991). To make matters even more confusing, Lopez-Neyra proposed synonymizing several African *Raillietina* spp., North American *Raillietina bakeri* and *Inermicapsifer* spp. into *R. madagascariensis* and placed *R. celebensis* in a novel genus *Meggittia*, a view rejected by most subsequent authors (López-Neyra, 1950, 1954) (Table 4).

Overall, it appears ‘*R. madagascariensis*’ likely represents a number of misidentified *Raillietina* spp., including several probable examples of *R. celebensis*. While the term *R. madagascariensis* appears to have fallen out of favour, a contemporary case report of ‘*R. madagascariensis*’ surfaced and it is not clear from the details provided whether the authors are treating this as a synonym of *Inermicapsifer madagascariensis* or as a *Raillietina* sp. proper (Prosl, 2005). Many other so-called *R. madagascariensis* cases may also represent *I. madagascariensis.* The latter misidentification probably stems from a failure to recover scolices and subsequent examination of rostella for hooks, as proglottids can appear similarly (particularly gravid proglottids where genital organs may be obscured by numerous egg capsules).

One other species besides *R. celebensis* has been described from human infections in Asia. Chandler and Pradatsundarasar (1957) provide a detailed description of multiple specimens reportedly in good condition recovered from two children in Thailand, and found similarities to published reports of *R. formosana*, *R. celebensis* and *R. madagascarensis*. However, the size of the eggs was much larger than typical *Raillietina* species (100–115 × 38–42 *μ*m *vs* 34–60 × 20–45 *μ*m), leading them to propose a new species *R. siriraji.* Thus far, this name appears to be regarded as valid (Matevosyan, 1966), although it is listed as a synonym of *R. celebensis* by Fain *et al*. (1977).

New World case reports are fewer in number than those from Asia and Pacific island nations, and all have been attributed to *R. demerariensis* and its various synonyms (Table 4) (Belding, 1965). While many authors agree that synonymy is appropriate, there has been some argument that the variations among described neotropical *Raillietina* spp. are more significant than the Old World and that lumping should be approached with caution (Chandler and Pradatsundarasar, 1957).

The sole report of *Raillietina* in a human from the USA is the most recent at the time of writing, and species identity was not determined. A child in Hawaii passed proglottids containing egg capsules that were repeatedly misidentified as *Dipylidium*, until re-examined at a reference laboratory and identified as being those of *Raillietina* sp. (Davis *et al*., 2019). Few other morphological details were available for subgeneric or specific determination and no scolex was recovered to definitively rule out *Inermicapsifer* – although the child did not have travel history to Cuba or Sub-Saharan Africa, so *Raillietina* sp. is most plausible. The authors suggest that this could represent infection with a chicken-associated *Raillietina* (e.g. *R. tetragona*), as free-ranging chickens are common in the area. However, all previous reports of zoonotic *Raillietina* involve rodent-infecting species. The introduced Polynesian rat (*R. exulans*) is a natural host for *R. siriraji* (Roberts, 1991), so this cannot be excluded as a possible reservoir host for a known zoonotic *Raillietina* in Hawaii.

The failure to recover scolices from patients passing proglottids remains a large roadblock in specific identification, and even if scolices are available, interpretation of some features may vary. For example, Margono *et al*. (1977) reported that viewing rostellar hooks directly or obliquely on the lateral side influenced their appearance enough to appear between *R. formosana* or *R. garrisoni* (although now both are regarded as synonyms of *R. celebensis*). Other factors such as the quality of fixation and anthelmintic treatment may further distort morphology – the former of which is to blame for much of the historical debate over ‘*Taenia madagascariensis*’. There is obviously a great need to revisit classical morphological examination and augment these with modern molecular studies on potentially zoonotic *Raillietina* spp. to more definitively resolve long-standing taxonomic issues and elucidate the true number of species and their distribution. These sorts of studies would also be useful in the re-evaluation of the sprawling *Raillietina* genus as a whole.

*Clinical and epidemiological characteristics*: While undoubtedly an uncommon event, it is difficult to assess the frequency of zoonotic *Raillietina* spp. infections with confidence owing to nomenclatural confusion and potential misidentifications with other cestodes. Cases attributed to *R. celebensis* have been described most commonly from Southeast and East Asia (Indonesia, Malaysia, Vietnam, Thailand, Myanmar, Taiwan, Japan) and the Philippines (Bonne and Mreyen, 1940; Miyazaki, 1950; Baer and Sandars, 1956; Pradatsundarasar, 1960; Fain *et al*., 1977; Margono *et al*., 1977). A plurality of reports exist from French Polynesia, including the islands of Tahiti and Mo'orea, although this may not truly represent a focus of infection but rather may reflect an increased awareness of clinical investigators in that region (Fain *et al*., 1977; Rougier *et al*., 1980, 1981). Sporadic cases in humans and rats have also been reported from Australia (Queensland) and India (Baer and Sandars, 1956; Niphadkar and Rao, 1969). The endemic range of this cestode may have expanded into the New World as, in 2017, *R. celebensis* was reported from Brazil during a survey of *R. norvegicus* (de Oliveira Simões *et al*., 2017). Detailed morphometrics reported in this case were consistent with *R. celebensis*, although the 18S gene sequences generated were not useful in definitively confirming species based on existing sequences (which are mostly from avian *Raillietina* spp.). This again underscores the need for careful molecular characterization of this group, to more accurately determine if this species has truly been introduced to the New World or if this represents a new (or existing) neotropical *Raillietina.*

*Raillietina demerariensis* is believed to represent the primary species infecting humans in the New World, although reports are scant compared to *R. celebensis* and its geographic occurrence has not been recently studied. Zoonotic infections have been recorded primarily from Ecuador, and to a lesser extent British Guyana, Cuba and Honduras (Dollfus, 1939; Joyeux and Baer, 1949; Belding, 1965). The occurrence in rodents is broader and includes Venezuela and Suriname, and numerous species reported as synonyms of *R. demerariensis* have been identified across the West Indies (Dollfus, 1939; Joyeux and Baer, 1949; Sato *et al*., 1988).

The final known zoonotic species, *R. siriraji*, has only been described from Thailand (Chandler and Pradatsundarasar, 1957; Pradatsundarasar, 1960; Charoenlarp and Radomyos, 1973; Chenchittikul *et al*., 1983), although a re-evaluation of morphometrics from other *Raillietina* spp. occurring in Southeast Asia is necessary to determine if this species is restricted to Thailand, or if it occurs more broadly. Many published studies do not report sufficient details to make this determination, and surveys of rodents often simply report ‘*Raillietina* sp.’

Nearly all reports in humans are among children, mostly under 3 years of age (Chandler and Pradatsundarasar, 1957; Pradatsundarasar, 1960; Rougier *et al*., 1980, 1981). The presumed exposures are *via* accidental (or intentional) consumption of infected arthropod intermediate hosts. A case series of *R. siriraji* infections in Thai children highlights that seven out of nine cases had a history of putting cockroaches in the mouth (Pradatsundarasar, 1960). The cockroach has yet to be definitively confirmed as an intermediate host for *R. siriraji*, but it seems plausible based on other *Raillietina* spp. Other details on the clinical history and potential routes of exposure among infected children are scanty.

Given the scarcity of case reports, it is difficult to make assumptions about the pathogenicity and true clinical spectrum of *Raillietina* infections. While likely that most infections are asymptomatic as typical for intestinal cestodiases, many (but not all) existing case reports involve symptomatic patients. Episodic diarrhoea and loose stools are frequently described, although it is difficult to unambiguously establish causality due to other potential enteric infections in the endemic tropical regions (Chandler and Pradatsundarasar, 1957; Rougier *et al*., 1981). Other symptoms are non-specific and include abdominal discomfort and distention, poor appetite and irritation. Of note is that in veterinary medicine, *Raillietina* and the closely related *Davainea* (which formerly included some of the human-infecting *Raillietina* species) are considered the most pathogenic cestode genera infecting poultry, known to cause substantial intestinal pathology and associated complications, including nodule formation (Horsfall, 1938; Dunn, 1978). Though not necessarily applicable to humans, this demonstrates the pathogenic potential of the genus, particularly if heavy infections are acquired.

Identification and diagnosis to genus level can generally be achieved by morphology owing to the unique appearance of *Raillietina vs* other human-infecting cestodes. Dissection or forced expression of egg capsules from proglottids should immediately identify the material as belonging to either *Raillietina* or *Inermicapsifer.* If *Raillietina* infection is suspected, every effort should be made to examine the important features of egg capsules and the genital pore of proglottids, particularly for distinguishing proglottids from *Inermicapsifer.* Scolices provide an unequivocal distinction between these two groups (CDC-DPDx, 2019), though as with many cestode infections, these are not often recovered due to their small size. Though molecular studies are generally lacking for zoonotic *Raillietina* spp., PCR and sequencing on recovered material could possibly be useful for confirmation of genus. Treatment is not standardized due to the rarity of cases, though single-dose praziquantel has proven effective (Davis *et al*., 2019).

### Inermicapsifer

#### Taxonomy and morphology

**Fig. 3.**
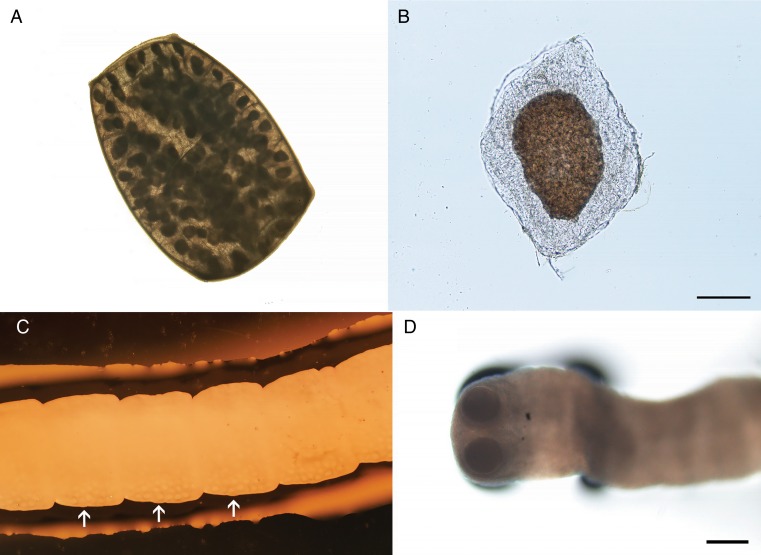
Specimen of *Inermicapsifer madagascariensis* from Cuba. (A) Gravid proglottid (4 mm long); (B) egg capsule liberated from gravid proglottid (scale bar = 100 *μ*m); (C) portion of strobila, showing median genital pores (arrows) (~30 ×  magnification); (D) unarmed scolex (scale bar = 200 *μ*m). Photos courtesy of DPDx, Centers for Disease Control and Prevention.

*Inermicapsifer* is a genus within Anoplocephalidae that includes parasites primarily of rodents, hyraxes and pangolins. This genus is similar in appearance to *Raillietina* in many respects apart from the scolex, which is unarmed (0.40–0.55 mm) with deep, cup-shaped suckers (Fig. 3). For the single accepted zoonotic species (*I. madagascariensis*), the maximum length is generally reported around 70–420 mm, varying by the degree of fixation, with around 300–360 segments in fully developed specimens. The proglottids are trapezoidal and prominently craspedote, and mostly wider than long, except for gravid ones which take on a more barrel-shaped appearance similar to *Raillietina*. For *Inermicapsifer* spp., the genital pore is mostly unilateral but some specimens may show an irregularly alternating pattern. In *I. madagascariensis*, the genital pore is situated in the middle point of the lateral margin. The genital ducts pass between osmoregulatory canals. The testes are numerous (usually 48–55 per mature proglottid) and a greater proportion are situated mostly on the aporal side of the female glands. The ovary is fan-shaped (~0.2 mm wide), with the vagina posterior to the small cirrus pouch (0.10–0.15 mm). The uterus in younger segments is visible as a branching structure, but in most segments, it breaks down into egg capsules each containing several eggs (Schmidt, 1986). Mature proglottids shed in feces are replete with such egg capsules, usually around 100–125, which create a mosaic or reticulated pattern (Fig. 3) reminiscent of *Raillietina.* Like *Raillietina*, the egg capsules are polygonal in shape and have a dark inner portion and a parenchymatous outer portion (Fig. 3). Each egg capsule generally contains 8–15 eggs, though sometimes fewer. The genital pore is not easily visible in very gravid proglottids (Baylis, 1949; Baer *et al*., 1950; Baer, 1956).

The taxonomic treatment of the genus *Inermicapsifer* has never been conclusively established and has been debated for decades. When first established by Janicki in 1910, the genus *Inermicapsifer* was placed in the subfamily Linstowiinae within Anoplocephalidae (Beaver *et al*., 1984). Originally proposed by Lopez-Neyra, it was later transferred by Spasski to a new subfamily Inermicapsiferinae on the basis of egg capsules containing multiple eggs, and the position of the ovary in the poral half part of the proglottid (Mettrick and Weir, 1963; Khalil *et al*., 1994). This subfamily is reflected in widely-used cestode identification texts (Yamaguti, 1959; Schmidt, 1986; Khalil *et al*., 1994), but its validity has not been universally recognized (Baer and Fain, 1955; Stunkard, 1961; Mettrick and Weir, 1963). It should also be noted that apart from the unarmed scolex, *Inermicapsifer* seems to share more morphological characteristics with *Raillietina* and other Davaineidae (e.g. uterine development, nearly identical egg capsules, egg morphology) than it does with classical Anoplocephalidae, leading to arguments for its inclusion in Davaineidae instead (Baylis, 1949; Baer and Fain, 1955; Stunkard, 1961; Mettrick and Weir, 1963). However, a familial characteristic of Davaineidae is an armed scolex with hammer-shaped hooks, thus precluding the placement of *Inermicapsifer* in the family as it stands now (Khalil *et al*., 1994). In the future, perhaps with supporting molecular phylogenetic studies, the diagnostic features defining these relevant taxa may be further evaluated and revised.

#### Life cycle and hosts

The life cycle is not completely known, but it probably does not differ substantially from *Raillietina* and other similar cestodes that involve an intermediate host with a cysticercoid infective to the definitive host (Fig. 1). In contrast to the zoonotic *Raillietina* spp. which are predominately parasites of the two peridomestic rats (*R. norvegicus* and *R. rattus*), *I. madagascariensis* is very seldom reported from peridomestic rats and instead is a parasite of native African rodents and hyraxes (Goldsmid and Muir, 1972). The two most commonly reported hosts in endemic regions of Sub-Saharan Africa are the Natal multimammate rat (*Mastomys natalensis*) and Gambian pouched rat (*Cricetomys gambianus*) (Hira, 1975). Other host species in which *I. madagascarensis* has been found include the rock hyrax (*Procavia capensis*), red rock rat (*Aethomys chrysophilus*), black-tailed tree rat (*Thallomys nigricauda*), the South African pouched mouse (*Saccostomus campestris*), the South African vlei rat (*Otomys irroratus*), the silvery mole-rat (*Heliophobius argentocinereus*) and grass rats (*Arvicanthus* spp.) (Ortlepp, 1961). A natural infection in a captive-bred chinchilla (*Chinchilla* sp.) was also reported (Goldsmid and Muir, 1972).

An important point is that *I. madagascariensis* has never been found in free-ranging animal definitive hosts in island nations, including Cuba, Madagascar, Mauritius and Comoros (Fain, 1950; Baer, 1956; Hira, 1975). Some authors have suggested that this indicates adaptation to humans as a reservoir in the absence of suitable rodents (Kourí, 1944; Hira, 1975; González Núñez *et al*., 1996). However, no genetic studies on *Inermicapsifer* exist and there are no sequences available in GenBank at the time of writing. It is possible that genetic differences may exist between rodent-adapted and human-adapted strains. This pattern has been observed in other helminths classically considered zoonotic, such as the nematode *Oesophagostomum bifurcum* (Gasser *et al*., 2006). The sole finding of *I. madagascariensis* in animal hosts in Cuba is a single report of incidental infections in laboratory-reared ‘white rats’; the route of exposure is unknown nor has there been further evidence for the existence of a zoonotic cycle (Kourí and Kourí, 1952).

The identity of the intermediate host is not known. Various authors have proposed terrestrial arthropods, including ants, beetles and mites (Ortlepp, 1961). Whether or not oribatid mites are involved is an important question from a taxonomic point of view. If they are not competent intermediate hosts, the argument for retaining *Inermicapsifer* in Anoplocephalidae becomes weaker, as nearly all members of said family require oribatid mite intermediate hosts and this is considered an important taxonomic feature. *Inermicapsifer* spp. egg capsules are much larger than typical anoplocephalid eggs, which are passed singly, and may prove too large for small oribatid mites to ingest (Baylis, 1949). On the other hand, the capsules are similar in size and character to those of *Raillietina* spp., which are known to infect ants and beetles as intermediate hosts.

#### Human infections

*Identity and nomenclature of zoonotic species*: Only one valid species is recognized in zoonotic infections, *I. madagascariensis*, but the history of this name and species is fairly involved for the same reasons as the zoonotic *Raillietina* spp. In 1938, Kourí initially described specimens from human patients in Cuba as *R. cubensis*. Upon further examination of the unarmed scolices, these specimens were determined not to be *Raillietina*, so they were re-described as *Inermicapsifer cubensis* (González Núñez *et al*., 1996). Fain (1950) later demonstrated that *Inermicapsifer arvicanthidis* from African rodents was identical to all existing descriptions of *I. cubensis*, a sentiment also reflected earlier by Baylis (1949) in the first human case report from continental Africa (Kenya). Within 6 years after the synonymizing of *I. cubensis* with *I. arvicanthidis*, during an investigation into the taxonomically ambiguous ‘*T. madagascariensis*’, Baer (1956) proposed the new name *I. madagascariensis* to unite these names and four other synonyms (while some others reported under the name *T. madagascariensis* were determined to represent *R. celebensis*). No other *Inermicapsifer* spp. (e.g. *I. guineensis*, *I. hyracis*) have been identified in human infections.

*Epidemiological and clinical aspects*: Endemic to the disparate locations of Sub-Saharan Africa, the Malagasy region and the West Indies, it is not entirely clear where *I. madagascariensis* originated. Generally, it is believed that the parasite was brought from Africa to the West Indies *via* the African slave trade, and perhaps to Madagascar and peripheral islands *via* Creole labourers associated with French settlers (Baer, 1956; Goldsmid and Muir, 1972). In continental Sub-Saharan Africa, *I. madagascariensis* appears to be broadly distributed and cases have been reported from Kenya, Rwanda, Burundi, South Africa, Zimbabwe, Malawi and Zambia (Baylis, 1949; Ortlepp, 1961; Goldsmid, 1964; Nelson *et al*., 1965; Goldsmid and Muir, 1972; Hira, 1974, 1975). Sporadic records of human infections also exist across the East African Indian Ocean islands (Madagascar, Mauritius, Comoros and Reunion Island) (Bailenger and Carcenac, 1970). Natural infections in rodents have also been found in Senegal though no human cases have been reported from West Africa (Sall-Dramé *et al*., 2010). In a summary of seven cases from Zimbabwe over about 8 years, it is most common in children between 1 and 3 years of age, but also occurs in 4–5 years old children, similar to previous reports from East Africa (Goldsmid and Muir, 1972). Patients in published reports are almost always of European descent (Nelson *et al*., 1965; Goldsmid and Muir, 1972). Authors suggest that infections in African children go unreported by parents in rural Africa due to mild symptomology and/or a lack of awareness, and so the infection may be much more common than generally assumed (Nelson *et al*., 1965; Goldsmid and Muir, 1972).

In Cuba, a hundred or so human cases were reported by Kourí (1944) (as *I. cubensis*) primarily from Havana and surrounding provinces prior to 1948. Not until 1996 were cases identified and reported in literature again; since then, approximately 45 cases primarily from regions surrounding Havana and Santa Clara have been diagnosed (and reported under the correct name *I. madagascariensis*) (González Núñez *et al*., 1996; Mayor and Herrera, 2004; Herrera Valdés *et al*., 2007). Age ranges and symptomology of patients are generally parallel to those reported in Africa. Among nine cases diagnosed in a single San Jose hospital, all patients were under 3 years old (5/9 between 1and 2 years old), 7/9 were male and 7/9 were from rural regions of the province (Pérez *et al*., 2009). In every case, it was the passing of motile proglottids by children that prompted parents to seek clinical attention. Anorexia and diarrhoea were also reported in six and four of these cases, respectively; however, a few children also had *Giardia* coinfections (Pérez *et al*., 2009). Also similarly to the Sub-Saharan African cases, nearly all cases were diagnosed in children of European descent in these case series.

Nearly all contemporary reports of human *I. madagascariensis* infections originate from Cuba and not Sub-Saharan Africa, perhaps from a lack of awareness or research interest in the area. A report of ‘*Raillietina madagascariensis*’ from a child who had travelled to Mauritius likely represents a case of *I. madagascariensis* (Prosl, 2005). As discussed previously, this name is problematic, and it is not clear whether or not the authors intended this as a synonym of *I. madagascariensis* or as a ‘true’ *Raillietina* sp. The proglottids were identified as belonging to *Raillietina* using Miyazaki's textbook *Helminthic Zoonoses*, which covers only *R. celebensis* and not *Inermicapsifer* (Miyazaki, 1991), and then used a key of Lopez-Neyra (which retains *R. madagascarensis* as a valid name and regards *Inermicapsifer* spp. as belonging to *Raillietina*) to identify species. The authors come to the conclusion that these proglottids are not *R. celebensis* due to the number of eggs per capsule, but the reported number (5–10 eggs/capsule) is more typical of *I. madagascarensis* (Hira, 1975). Importantly, the proglottid shown has a genital pore on the median point of the lateral margin (also typical of *Inermicapsifer*) and not in the anterior portion (as typical of human-infecting *Raillietina* spp.). As with many cases, the scolex was not recovered, precluding unequivocal identification of this cestode. This problem highlights the need for definitive clarification of taxonomy and nomenclature.

The source of infection is not entirely clear as the intermediate host is not known, though the predominance of young children is similar to other cestodiases transmitted *via* ingestion of arthropods (e.g. hymenolepiasis, bertielliasis, dipylidiasis). Nelson *et al*. (1965) note that all cases recorded outside of Africa have been in regions that rely heavily on sugar cane farming, where eating raw cane is a common habit that could lead to accidental ingestion of arthropods. However, it is also suggested that very young children and infants are unlikely to become infected this way as they may be unable to chew raw cane (Hira, 1975).

Most cases do not involve severe symptoms, and presentation is the typical mild collection of symptoms associated with most intestinal cestodiases, or are asymptomatic. Reported symptoms include irritability, abdominal pain, anorexia, gastrointestinal distress and generalized malaise (Hira, 1974; Pérez *et al*., 2009). Like many of the agents discussed in this review, it is the presence of motile proglottids (often mistaken for rice grains) in the stool that prompts clinical attention. A few cases note weight loss in children which ceased following treatment, suggesting perhaps this was associated with *I. madagascariensis* infection and not simply other causes in endemic areas (Goldsmid and Muir, 1972).

Diagnosis is based on the examination of the rice grain-like shed proglottids; since egg capsules are released passively after the disintegration of the proglottid, direct smears or flotation of recently-collected patient stool samples will not reveal free egg capsules (Hira, 1974). For the proper identification of *I. madagascariensis*, care must be taken to distinguish proglottids from those of *Raillietina* spp. The scolex provides the easiest distinction (unarmed *Inermicapsifer vs* armed *Raillietina*), but as discussed throughout, this is seldom recovered from human infections. The position of the genital pore is another useful characteristic (middle of lateral margin for *Inermicapsifer*; anterior quarter for *Raillietina*), but this can be difficult to visualize on very gravid proglottids and may require staining (Goldsmid and Muir, 1972). If egg capsules are liberated from proglottids, the number of eggs per capsule may also provide a diagnostic clue (generally 8–15 eggs for *Inermicapsifer*; 1–4 for known zoonotic *Raillietina*). Treatment is straightforward using cestodicidal agents such as niclosamide and praziquantel (Goldsmid and Muir, 1972; Mayor & Herrera, 2004). Similar to *D. caninum*, benzimidazoles appear to lack efficacy; in one case, a child with concurrent *E. vermicularis* infection was treated with thiabendazole, with no effect on the *Inermicapsifer* tapeworm (Goldsmid and Muir, 1972).

### Mesocestoides

#### Taxonomy and morphology

Mesocestoididae is an unusual family within Cyclophyllidea, sharing some characteristics with Pseudophyllidean tapeworms. The family contains only two subfamilies, both of which are monogeneric – *Mesocestoides* is the only genus within Mesocestoidinae (Wardle and McLeod, 1952). Infecting a variety of terrestrial mammalian carnivores, adults are long and slender, measuring about 30–100 cm with a maximum width of 2.5 mm, typically with 600–1000 proglottids at maturity. Proglottids towards the anterior portion of the strobila are somewhat wider than long, and with maturity becoming more long than wide with convex margins (Miyazaki, 1991). Testes are spherical, 20–40 *μ*m in diameter and usually numbered around 60–80 in specimens recovered from humans (Miyazaki, 1991; Fuentes *et al*., 2003). The uterus is a simple blind tube aligned vertically in the median portion of the proglottid (Wardle and McLeod, 1952). The genital pore does not open on the lateral margin of the proglottid as typical for most Cyclophyllidea; it is located in the centre of the proglottid on the ventral surface, similar to some Pseudophyllidea. The scolex is unremarkable, having four large suckers and no rostellum (Wardle and McLeod, 1952).

The most salient feature of *Mesocestoides* is the possession of a well-developed parauterine organ, appearing as a dense circular to pear-shaped structure (250–300 *μ*m in diameter) in the posterior portion of the proglottid. This was once considered to be a ‘true’ parauterine organ in contrast to the structures of the same name in some anoplocephalid cestodes, as it was believed to arise independently of uterine tissue (Chandler, 1946; Wardle and McLeod, 1952; James, 1968). However, this assumption was based on the developmental studies using light microscopy and limited specimens, which may be challenging to interpret (Conn, 1987). Ultrastructural analysis of the parauterine organ and the uterine tissues have since confirmed a relationship between the uterine epithelium and the development of the parauterine organ (Conn, 1987). This structure is fully developed in aged proglottids; further down the strobila, eggs move from the uterus into the parauterine organ, which becomes swollen and enlarged in terminal segments. At this point, the uterus will appear empty and the testes degenerate completely (Miyazaki, 1991).

#### Life cycle and hosts

**Fig. 4.**
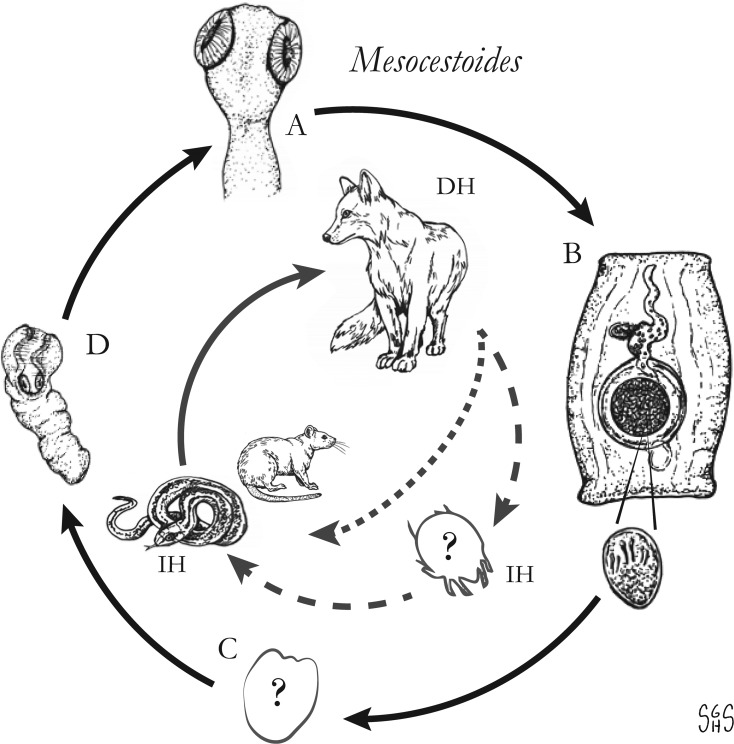
Proposed life cycle schemes for *Mesocestoides* spp., showing two-host (dotted line) and three-host (dashed line) hypotheses. Cestode stages shown on the outside: (A) scolex of an adult; (B) gravid proglottids with oncosphere; (C) unknown cysticercoid or first larval stage; (D) tetrathyridium; representative definitive host (DH) and intermediate hosts (IH) on the inside (Drawings by SGH Sapp).

The life cycle of *Mesocestoides* spp. is divergent from the more familiar Cyclophyllidea and our understanding remains incomplete. Though debated for some time, it is now generally accepted based on the experimental evidence that not one but two intermediate hosts are involved, although some recent field observations have called this back into question (Fig. 4) (Loos-Frank, 1991; McAllister *et al*., 2018). Proglottids are passed in the feces of the definitive host; they are highly motile and may crawl actively to the surface of the fecal deposit. The fragile oncospheres die within less than a minute if exposed after breakage of the proglottid, so transmission to the first intermediate host (see below) likely occurs by the ingestion of the entire intact proglottid. The density of the parauterine organ appears to provide some protection, as oncospheres remain motile for 25–40 days within the intact structure (James, 1968; Miyazaki, 1991).

The role of the first intermediate host and metacestode development therein is the missing link in the complete characterization of the *Mesocestoides* life cycle. The most popular hypothesis is that this host is an invertebrate that consumes proglottids/eggs found in feces of the infected definitive host, which then supports the development of the oncosphere into a poorly-understood metacestode stage, which probably resembles a procercoid based on *in vitro* development (Voge and Seidel, 1968; Loos-Frank, 1991). The assumption that an invertebrate serves as a first intermediate host is based both on life cycles of other cestodes and the observation that exposure of vertebrates inoculated with oncospheres/proglottids under many conditions has failed to establish any evidence infection on multiple occasions (Webster, 1949; James, 1968; Loos-Frank, 1991). This seems to suggest that there must be some invertebrate intermediate host despite the failure to identify it. Various authors have proposed terrestrial arthropods such as dung beetles, ants, roaches and mites as potential first intermediate hosts. A singular report of finding *Mesocestoides* ‘cysticercoids’ in oribatid mites has not been further substantiated in subsequent experimental studies and surveys (James, 1968). Attempts to infect about 50 different species of invertebrates, including several families of arthropods, annelids and mollusks, with the eggs of *Mesocestoides* spp. have consistently yielded negative results (Webster, 1949; James, 1968). *Mesocestoides* sp. DNA was detected in 3.1 and 2.4% of *Lasius niger* and *Tapinoma sessile* ants, respectively, collected from San Miguel Island (California). However, attempts to infect mice with such ants were not successful (Padgett and Boyce, 2004). Of note, a recent report describes the stages interpreted as transitional, pre-tetrathyridial stages in the body cavity of a ground skink (*Scincella lateralis*), suggesting that development from the hexacanth embryo to tetrathyridium could possibly be taking place within a single vertebrate host (McAllister *et al*., 2018). As it stands, the number of hosts involved in the *Mesocestoides* life cycle remains an outstanding question and the complete life cycle has yet to be demonstrated experimentally.

The second metacestode stage, or tetrathyridium, somewhat resembles a sparganum but has an invaginated, four-suckered scolex (Nelson *et al*., 1965). It is a solid structure without cystic characteristics, except under aberrant conditions (James, 1968). The second intermediate host presumably becomes infected *via* consumption of the unknown first intermediate host, which leads to the development of the tetrathyridia in various sites (commonly the peritoneal cavity, mesenteries, also in visceral organs). The host specificity demonstrated by *Mesocestoides* tetrathyridia is remarkably low, having been recovered from a vast array of mammals, birds, reptiles and amphibians (Miyazaki, 1991).

On rare occasions in nature, tetrathyridia become asexually proliferative which can lead to heavy disseminated infections and death. Instances have nearly all been ascribed to *Mesocestoides corti*, which was later reidentified as *Mesocestoides vogae* by Etges (1991). Reports of tetrathyridial asexual budding ascribed to other *Mesocestoides* spp. exist (Conn, 1990; Crosbie *et al*., 2000; Galán-Puchades *et al*., 2002; Conn *et al*., 2010), though precise species identification is challenging and the presentation varies widely. Some types of tetrathyridial asexual proliferation described involve in the division of the apical extremity resulting in multicephalic forms, budding of the hindbody, and the production of daughter metacestodes from a mother tetrathyridium (Galán-Puchades *et al*., 2002; Conn *et al*., 2010; McAllister *et al*., 2018). The factors influencing the development of these proliferative forms and the mechanism(s) are still not completely understood (Conn *et al*., 2010). A mechanism involving malignant transformation, also observed with *H. nana*, has been proposed (Conn *et al*., 2010; Conn, 2016). High-intensity infections following the ingestion of large numbers of oncospheres are prone to being inaccurately ascribed to asexual proliferation, thus this term should be applied only if abnormal characteristics (e.g. multicephalic or acephalic forms, external budding) can be demonstrated (Conn, 2016).

Definitive hosts are both wild and domestic carnivorous mammals, and adult *Mesocestoides* spp. may be prevalent in some wildlife populations. In North America, *M. variabilis* is a common finding in a variety of native mesopredators, such as the raccoon (*Procyon lotor*), opossum (*Didelphis virginiana*), striped skunk (*Mephitis mephitis*), spotted skunk (*Spilogale* spp.), lynx (*Lynx rufus*), coyote (*Canis latrans*), kit fox (*Vulpes macrotus*) and gray fox (*Urocyon cinereoargentus*) (Voge, 1955; James, 1968). Other New World *Mesocestoides* species [e.g. *M. vogae* ( *=* *corti*), *M. latus*, *M. kirbyi*] also occur across similar hosts. The primary host of European *Mesocestoides* spp. (*M. lineatus*, *M. litteratus* and unidentified *M. sp.*) appears to be the red fox (*Vulpes vulpes*), but infections occur in wolves (*Canis lupus*), European badgers (*Meles meles*) and raccoon dogs (*Nyctereutes procyonoides*) (Thompson, 1976; Jones *et al*., 1980; Moks *et al*., 2006; Bagrade *et al*., 2009; Hrčkova *et al*., 2011; Bružinskaitė-Schmidhalter *et al*., 2012). Surveys of Asian wild definitive hosts are less extensive. In Japan, infections in raccoon dogs and Japanese martens (*Martes melampus*) have been reported (Sato *et al*., 1999). Wolves, red foxes, corsac foxes (*Vulpes corsac*) and snow leopards (*Panthera uncia*) were recently identified as *Mesocestoides* sp. hosts in Mongolia (Ulziijargal *et al*., 2020).

As the first intermediate host is unknown and attempts to infect arthropods have never been successful, what is known regarding tetrathyridial and adult stage infections is entirely based on experimental infections involving transferring tetrathyridia derived from naturally-infected hosts among laboratory animals. Prepatent periods following exposure to tetrathyridia have been shown to be highly variable and are probably mostly due to variations in host competence, but inherent species-level differences cannot be ruled out. For example, a range of 16–52 days have been observed for experimentally-infected raccoons, but may as long as 80 days in domestic dogs and over 100 days in domestic cats (James, 1968). Shedding of proglottids may be irregular and vary seasonally (Skarbilovitch, 1945).

Some definitive host species may also develop tetrathyridial infections as second intermediate hosts. This is exemplified by domestic dogs, which may harbour intestinal infections with adult *Mesocestoides*, or develop potentially life-threatening invasive infections with tetrathyridia (canine peritoneal larval cestodiasis) (Eckert *et al*., 1969; Speckmann and Webster, 1975; Boyce *et al*., 2011). Domestic cats have also been shown to serve as both definitive and second intermediate hosts (Mueller, 1930; Eleni *et al*., 2007).

#### Human infections

*Identity of zoonotic species*: The only named species reported from human infections are *M. lineatus* in Asia and *M. variabilis* in North America. However, interspecific morphologic plasticity along with other factors complicates specific diagnosis and suggests that current taxonomic divisions in this genus are unsound. Even after examining many specimens from several definitive hosts, Voge (1955) considered these species impossible to distinguish unless the geographic origin was known, and remarked that appearance was greatly influenced by staining methods. Differences in vitellaria and number of testes for *M. variabilis* were described across its many carnivore hosts. The state of fixation (relaxed *vs* contracted) was also observed to greatly influence the appearance of some internal structures, including the shape of the parauterine organ and caudal appendage (James, 1968). Further morphological differences in the diameter of the testes, vitellaria, ovaries, position of the cirrus pouch and more are also apparently influenced by age of the proglottids and duration of infection (James, 1968). Differences between the presumed zoonotic species and other sympatric species occurring in natural definitive hosts (e.g. *M. latus vs M. variabilis*) are also reportedly very slight and subject to intraspecific variation and processing artefacts (Voge, 1955). Given that clinical specimens are typically not recovered under ideal conditions (e.g. brought in by patients after the expulsion, not relaxed prior to fixation, etc.), one may expect that an accurate species identification would be impossible under such circumstances.

Various molecular phylogenetic studies have attempted to further understand species delimitation in *Mesocestoides*; however, the genus is still far from a formal modern revision. In studies of *Mesocestoides* spp. isolates from the Western USA, three monophyletic clades were detected (A, B and C) among various hosts including dogs, coyotes, island foxes (*Urocyon litteratus*) and naturally-infected deer mice (*Peromyscus maniculatus*) (Crosbie *et al*., 2000). Morphological assessment of these defined clades revealed extensive overlap in measured characteristics (Padgett *et al*., 2005). Clade B specimens were most similar to *M. vogae* (which is possibly synonymous with *M. variabilis*), but clade A and C specimens could not be matched unambiguously to any existing *Mesocestoides* species description. None of the three clades was found to be host-specific, although only canid definitive hosts were represented (Padgett *et al*., 2005). The European picture is clearer than the American one. Among European isolates, *M. litteratus* and *M. lineatus* have been found to be distinct species based on some morphological features (number of testes, length of cirrus sac, shape of parauterine organ) and on multiple loci (12s, cox1, nad1) (Literák *et al*., 2006; Hrčkova *et al*., 2011; Zaleśny and Hildebrand, 2012). However, studies on ‘*M. lineatus*’ in East Asia – where it has been reported from human infections – are lacking and thus its specific status and relation to other *Mesocestoides* spp. are unknown.

There is a clear need for additional *Mesocestoides* genetic studies as well as conventional comparative studies involving several aspects of complete specimens processed in the same manner. As mentioned, it seems likely that some described species are invalid, whereas other names may represent assemblages of cryptic species. Without further characterization from multiple isolates across different hosts and regions, it is probably most appropriate to report *Mesocestoides* spp. infections in humans as ‘*Mesocestoides* sp.’, particularly given that recovering entire specimens from human infections is nearly impossible and the division between *M. lineatus* and *M. variabilis* may simply be a geographic assumption at present.

*Epidemiology and clinical characteristics*: Only about 27 cases of human intestinal infections have been reported; those accessible in English language or translatable medical literature are summarized in Table 5. Over half of these cases are from East Asia (mostly from Japan, fewer from Korea and China) and the remainder from the USA (California, Texas, Missouri, Mississippi, Louisiana, Ohio, New Jersey). Singular reports also exist from Rwanda and a Greenlander living in Denmark, though the latter is based on a personal communication and is not available as a standalone report (Fuentes *et al*., 2003).

**Table 5.** Summary of *Mesocestoides* spp. infections reported from humans

Location	Species reported	Age/sex	Possible exposure	Source
USA (Texas)	*M.* sp.	2 yo F	Unknown	CDC-DPDx (unpub. data) 2010
USA (Louisiana)	*M. variabilis*	19 mo M	Cajun sausage with game viscera	(Fuentes *et al*., 2003)
USA (Missouri)	*M. variabilis*	5 yo M	Unknown	(Gleason and Healy, 1967)
USA (California)	*M.* sp.	22 mo F	Unknown; exotic pet contact and presence of lizards in playground noted	(Schultz *et al*., 1992)
USA (Mississippi)	*M.* sp.	17 mo M	Unknown; noted numerous pets and livestock at home	(Hutchison and Martin, 1980)
USA (New Jersey)	*M.* sp.	14 mo M	Unknown; geophagy noted	(Gleason *et al*., 1973)
USA (Texas)	*M. variabilis*	13 mo F	Unknown	(Chandler, 1942)
Japan (Hamamatsu)	*M. lineatus*	51 yo F	Raw snake liver	(Ito *et al*., 1962)
Japan (Nagoya)	*M. lineatus*	42 yo M	Raw snake liver and blood, eel	(Morisita *et al*., 1964)
Japan (Tokyo)	*M. lineatus*	30 yo M	Raw snake blood, heart, and gall bladder	(Kamegai *et al*., 1967)
Japan (Tokyo)	*M. lineatus*	57 yo F	Raw snake blood, heart, and gall bladder	‘
Japan (Toyohashi)	*M. lineatus*	36 yo M	Raw snake and eel	(Kosaka, 1942)
Japan (Tokyo)	*M. lineatus*	31 yo M	Raw snake and snapping turtle	(Hagihara *et al*., 1964)
Japan (Saitama)	*M.* sp.	40 yo M	Raw snake	(Miyagi *et al*., 1965)
Japan (Gifu Prefecture)	*M. lineatus*	35 yo M	Raw snake liver and blood	(Ohtomo *et al*., 1983)
Japan	*M. lineatus*	46 yo M	Raw snake heart, liver, muscle, and blood	(Kagei *et al*., 1974)
Japan	*M. lineatus*	Unknown	Unknown	(Nagase *et al*., 1983)
South Korea	*M.* sp.	45 yo M	Raw snake meat	(Choi *et al*., 1967)
South Korea (Jeju-do)	*M. lineatus*	45 yo M	Raw chicken viscera	(Eom *et al*., 1992)
China	*M. lineatus*	Unknown	Unknown	(Fan, 1988)
China (Jilin Province)	*M. lineatus*	Unknown	Unknown	(Jin *et al*., 1990)
Denmark^a^	*M. variabilis*	Unknown	Unknown	Chandler; unpub. [noted in (Gleason and Healy, 1967)]
Rwanda/Burundi (Ruanda-Urundi)	*M.* sp. (nov?)	‘child’	Partridge meat	(Fain and Herin, 1954)

a ^a^ ‘Greenlander living in Denmark’.

Human mesocestoidiasis is generally regarded as a foodborne zoonosis, caused by the consumption of tetrathyridia in undercooked meat. An intriguing observation is the epidemiological split between known Asian and North American cases, regardless of the species validity questions discussed prior (Table 5). In the Japanese and Korean cases, patients are middle-aged adults, and nearly all were reported to have habitually consumed blood and organs of snakes (particularly *Elaphe quadrivirgata* and *Agkistrodon halys*, both known to harbour tetrathyridia) for perceived medicinal properties (Ito *et al*., 1962; Kamegai *et al*., 1967; Kagei *et al*., 1974). This contrasts with North American cases, which have all been documented in children aged 1–5 years (typically between 1 and 2 years). The source of infection is mysterious as raw/undercooked meat consumption is denied in all but one case. Details reported in some North American cases include contact with a variety of domestic animals, exotic pets and geophagy. However, these do not seem plausible sources for exposure to tetrathyridia, the only stage known to be infective to definitive hosts. Inoculation of laboratory mammals with *Mesocestoides* eggs *via* proglottids does not establish infection (Webster, 1949). If the theoretical arthropod first intermediate host is eaten, it would lead to a tetrathyridial tissue infection and not an adult intestinal infection based on the currently accepted life cycle scheme. Overall, it is difficult to say whether this confusion is a result of unreliable case histories, an incompletely understood life cycle, or some other factor, but the difference in the pattern of Asian and North American mesocestoidiasis remains striking.

As with most other intestinal cestode infections, infections can be asymptomatic and generally are not severe. Diarrhoea, intermittent abdominal pain, dizziness, poor appetite, weight loss, languor and cramping have been reported (Choi *et al*., 1967). It is the appearance of motile proglottids in the stool that has in nearly all cases prompted patients to seek clinical attention. The greatest burden ever detected in a human was in a case in Korea, where 32 strobilae were passed following treatment. The patient reportedly ‘habitually ate raw chicken viscera’, a habit which would explain the repeated exposure and accumulation of worms (Eom *et al*., 1992). The first described human case may have also represented a high burden; the patient reportedly expelled ‘about 35 feet of an unusual, very narrow type of tapeworm’ following administration of a vermifuge (Chandler, 1942). Intact strobilae recovered from human infections may measure from 30to 136 cm, often passed in long, continuous pieces post-treatment (Chandler, 1942; Choi *et al*., 1967; Kamegai *et al*., 1967).

Tetrathyridial infections have never been reported in humans. This possibility should not be entirely discounted, given the low host specificity and that tetrathyridial infections have been documented on multiple occasions in non-human primates (Fincham *et al*., 1995; Di Filippo *et al*., 2013; Tokiwa *et al*., 2014). Additionally, the superficial resemblance to spargana and other larval cestodes could possibly confound diagnosis.

Mesocestoidiasis, though rare, could possibly be underestimated and ought to be considered in cases where patients present with motile cestode proglottids and report raw or rare meat or viscera consumption. Diagnosis to genus level is straightforward for trained parasitologists, as the appearance of the proglottids with a true parauterine organ and surficial genital pore is unique to *Mesocestoides.* Since the proglottid morphology is so distinctive among known zoonotic cestodes, recovery of the scolex is not necessary for confirming identification. Treatment is simple and has been achieved with a variety of cestodicidal agents (e.g. praziquantel, niclosamide, paromomycin) (Gleason and Healy, 1967; Gleason *et al*., 1973; Hutchison and Martin, 1980; Fuentes *et al*., 2003).

## Conclusion

The diversity of cestodes that can infect humans is broader than generally appreciated – when properly examined, not every motile proglottid in the human stool will prove to be the typical *Taenia*, *Hymenolepis* or Diphyllobothriid. Considering the possibility of increasing exposures to rare zoonoses through modern factors such as travel and encroachment on ecosystems, it is likely that the incidence of human infections with these rare Cyclophyllidean tapeworms will increase in the coming decades. Among the unusual Cyclophyllidea discussed, many unanswered questions remain regarding basic taxonomy and nomenclature, life cycles and transmission, ecology in natural hosts and epidemiology in human cases. Modern genetic tools, especially if employed alongside classical morphological investigation, will certainly aid in resolving these issues, some of which have stood unaddressed for many decades. Improving the ‘visibility’ and raising interest among clinicians and laboratory staff will be critical in achieving these goals, as they are on the frontline of case recognition and the subsequent acquisition of material and clinical information from human cases necessary for further investigations. Prevention strategies – beyond proper food safety and sanitation practices – will be improved by a more complete understanding of the transmission and life stages of these parasites. Overall, improving our understanding of these infrequent human intestinal cestodiases will require collaboration between many parties, including morphologists, molecular parasitologists, clinicians, diagnostic laboratory staff, ecologists and the affected patients themselves.
